# Anatomy of the Bezymianny volcano merely before an explosive eruption on 20.12.2017

**DOI:** 10.1038/s41598-021-81498-9

**Published:** 2021-01-19

**Authors:** Ivan Koulakov, Pavel Plechov, René Mania, Thomas R. Walter, Sergey Z. Smirnov, Ilyas Abkadyrov, Andrey Jakovlev, Vesta Davydova, Sergey Senyukov, Natalia Bushenkova, Angelika Novgorodova, Tatyana Stupina, Svetlana Ya. Droznina

**Affiliations:** 1 Trofimuk Institute of Petroleum Geology and Geophysics SB RAS, Prospekt Koptyuga, 3, Novosibirsk, Russia 630090; 2 Novosibirsk State University, Pirogova 2, Novosibirsk, Russia 630090; 3 Institute of Volcanology and Seismology FEB RAS, Piip Boulevard, 9, Petropavlovsk-Kamchatsky, Russia 693006; 4 Fersman Mineralogical Museum RAS, Moscow, Russia; 5 Lomonosov Moscow State University, Geological Departments, Moscow, Russia; 6 GFZ German Research Centre for Geosciences, Telegrafenberg, 14473 Potsdam, Germany; 7 Sobolev Institute of Geology and Mineralogy SB RAS, Prospekt Koptyuga, 3, Novosibirsk, Russia 630090; 8 Kamchatkan Branch of Geophysical Survey RAS, Piip Boulevard, 9, Petropavlovsk-Kamchatsky, Russia 693006

**Keywords:** Geophysics, Petrology, Seismology, Volcanology, Natural hazards

## Abstract

Strong explosive eruptions of volcanoes throw out mixtures of gases and ash from high-pressure underground reservoirs. Investigating these subsurface reservoirs may help to forecast and characterize an eruption. In this study, we compare seismic tomography results with remote sensing and petrology data to identify deep and subaerial manifestations of pre-eruptive processes at Bezymianny volcano in Kamchatka shortly before its violent explosion on December 20, 2017. Based on camera networks we identify precursory rockfalls, and based on satellite radar data we find pre-eruptive summit inflation. Our seismic network recorded the *P* and *S* wave data from over 500 local earthquakes used to invert for a 3D seismic velocity distribution beneath Bezymianny illuminating its eruptive state days before the eruption. The derived tomography model, in conjunction with the presence of the high-temperature-stable SiO_2_ polymorph Tridymite in juvenile rock samples , allowed us to infer the coexistence of magma and gas reservoirs revealed as anomalies of low (1.5) and high (2.0) *Vp/Vs* ratios, respectively, located at depths of 2–3 km and only 2 km apart. The reservoirs both control the current eruptive activity: while the magma reservoir is responsible for episodic dome growth and lava flow emplacements, the spatially separated gas reservoir may control short but powerful explosive eruptions of Bezymianny.

## Introduction

Strong explosive volcanic eruptions can potentially affect population, transportation and infrastructure^1^. In recent decades, monitoring techniques and understanding of eruption precursors significantly advanced developments of empirical and probabilistic eruption forecasts^2, 3^. However, the used methods are often based on a set of sparse observations too far from the source, and are biased by subjective decisions of the involved experts. One of the most widely used concepts for eruption monitoring and early warning is based on the assumed presence of magma reservoirs in the upper crust (up to 5 km depth), causing localized inflation and characteristic pre-eruptive changes in seismicity^4, 5^. In this context, it is key to understand the physical mechanisms behind the observed phenomena in order to reveal the actual processes taking place inside the volcano prior to eruptions.

The structure of a plumbing system beneath an active volcano and its temporal evolution can be studied by various methods including seismic tomography^6–8^, remote sensing^9, 10^, and petrological analysis of rocks^11, 12^. Examples from other dome-building volcanoes suggest the presence of shallow temporally active gas pockets^13^ that are active over days to weeks^14, 15^. Such gas sources located at or nearby extruding viscous and degassed magmas^16^ affect conduit pressurization and explosivity of the volcano^17, 18^. However, because strong explosive eruptions pass very quickly, it is exceptionally rare to install special equipment at the right time and close enough to explore volcanic processes associated with such eruptions. Only few months prior to the 20 December 2017 explosive eruption at Bezymianny volcano, Kamchatka, we were lucky enough to deploy in August 2017 a portable seismic network and time lapse cameras viewing onto the edifice from different perspectives. In addition, we regularly tasked high resolution TerraSAR-X spotlight satellite acquisitions. Thus, we were able to observe the preparation phase of this eruption by means of local and regional seismic stations, satellite radar imaging, time-lapse monitoring cameras, and petrological investigations from post-eruptive sampling of fresh eruptive products. Integrating all these data enables us to develop a comprehensive model of the volcano's interior, showing the state of the plumbing system during only a few days before the large eruption.

### Bezymianny volcano

**Figure 1:**
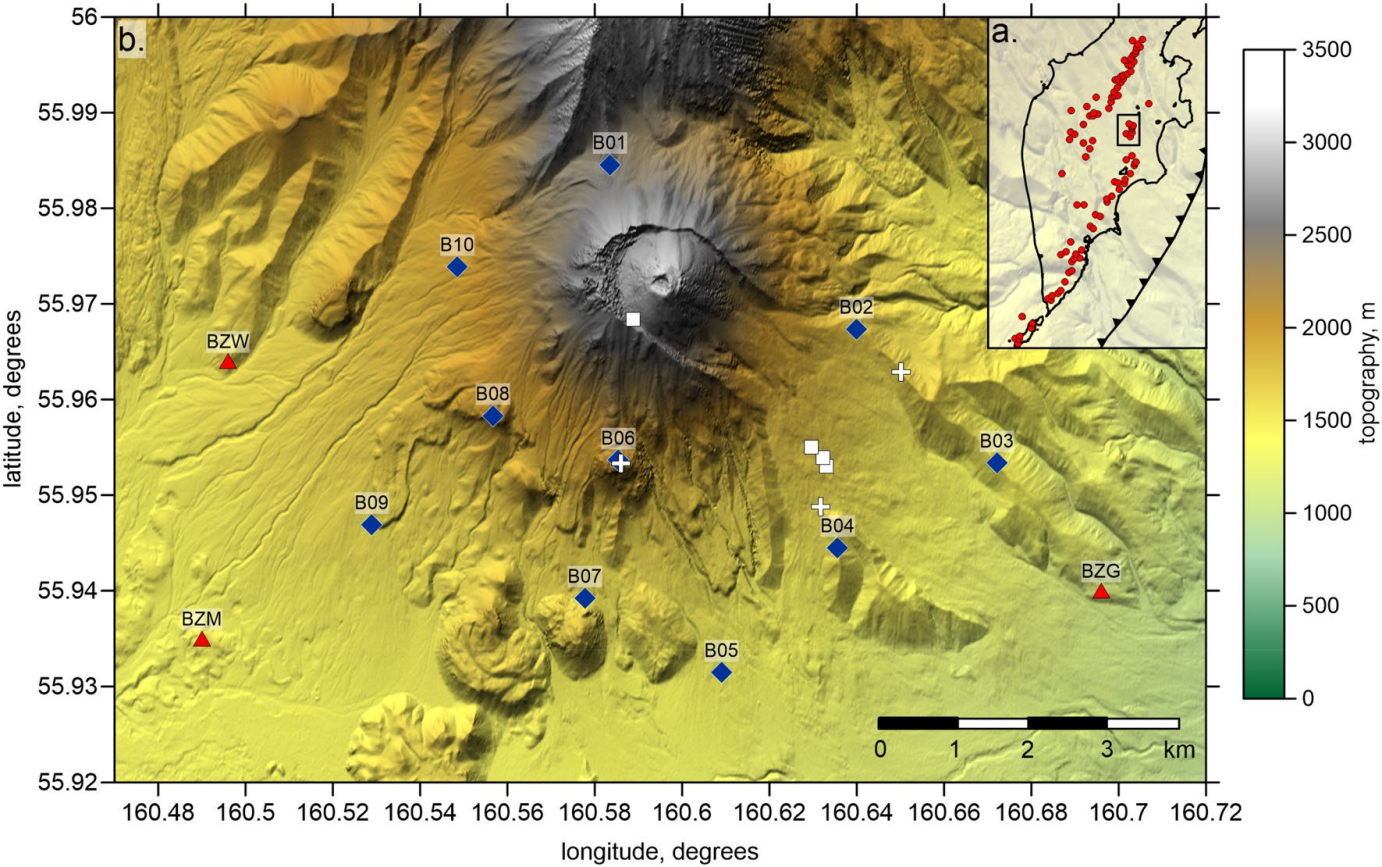
Study area. (**a**) Location of the study region (black rectangle) in the Kamchatka map. Red dots are the Holocene volcanoes. (**b**) Topography map in the area of the Bezymianny volcano (https://tandemx-science.dlr.de/). Red triangles depict permanent stations of the KBGS; blue diamonds are the temporary stations installed in 2017–2018, white squares are the rock sampling sites, and white crosses mark the locations of the time-lapse cameras. The image has been produced using the Surfer Golden Software 13 (https://www.goldensoftware.com/products/surfer).

**Figure 2:**
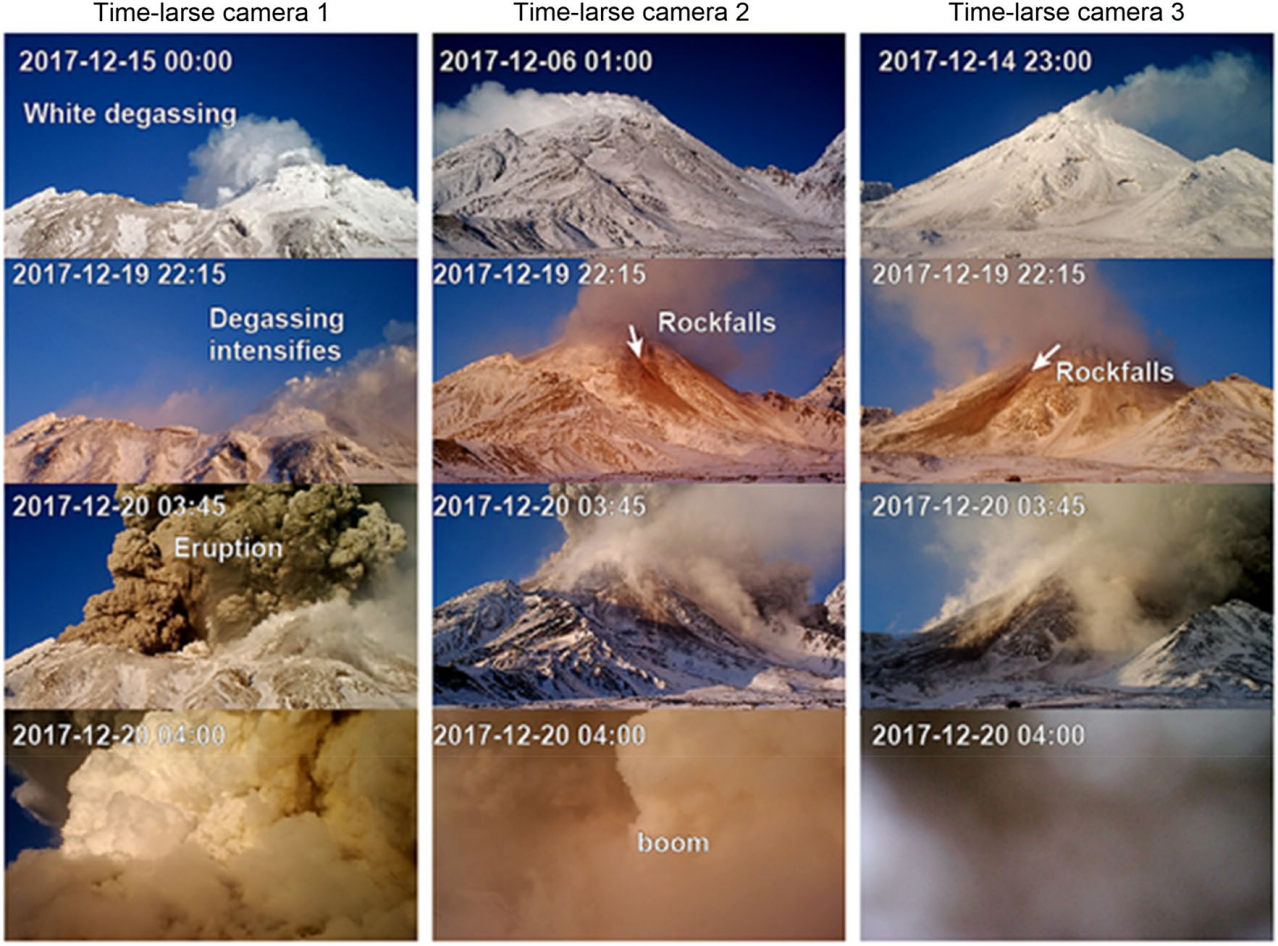
Images taken by three time-lapse cameras reveal visual signatures of the eruption preparation, such as rock falls and strong degassing 6 or more hours prior to the eruption. The lower row corresponds to the eruption moment. Date and time in UTC. Locations of the cameras are indicated by white crosses in Fig. 1.

Bezymianny is a basaltic-andesitic to dacitic dome-building stratovolcano^19, 20^ located within the Klyuchevskoy Group of Volcanoes (KGV) in Kamchatka near the northern edge of the Kuril-Kamchatka subduction zone (Fig. 1a). In 1956, the volcano produced a sector collapse together with a catastrophic lateral blast explosion^21–23^ renowned as one of the world’s strongest eruptions of the twentieth century. During the last decades, Bezymianny has been producing powerful but short-lived explosions one to two times per year^24^ and is therefore considered as one of the most active volcanoes in the world. All these recent eruptions occurred inside the 1956 sector collapse amphitheater, by today forming a steep central stratocone with a relative elevation of about 850 m (the total height of Bezymianny is 3020 m)^25^. One of the strongest recent eruptions at Bezymianny occurred on 20 December 2017^26^. Based on elevated rockfall activity in parts associated with exponentially increased seismicity rates, this event was anticipated by the Kamchatka Branch of Geophysical Survey (KBGS), which issued alert documents two days prior to the eruption^27^. Telemetric video cameras at distance operated by KBGS^28^ showed that this explosion was short (15–30 min) but powerful enough to produce an approximately 15-km-high ash plume (Fig. S1 of Supplementary) and deposited tephra over an area of 42,000 km^2^^26^. Visual signatures of the forthcoming eruption, such as rock falls and elevated degassing, can also be examined by three time-lapse cameras installed by us at the vicinity of Bezymianny (Fig. 2).

Based on the data of permanent seismic stations operated by the KBGS and several temporary seismic networks installed in the vicinity of Bezymianny and its neighboring volcanoes of the KGV, previous studies^29–35^ revealed multilevel magma storages in the crust and the uppermost mantle: the shallow-most level being located at circa 8 km depth. Multiple petrological studies^36, 37^ revealed consistent storage conditions of magmas for Bezymianny, but they could not yet characterize the lateral complexity and dimensions of potential reservoirs.

The upper part of the plumbing system beneath Bezymianny consequently remains poorly understood. In particular, since explosive eruptions of Bezymianny are accompanied by the release of extensive amounts of volcanic gases, the question arises from which level in the plumbing system these gases may originate. Here, we attempt to address this and other puzzles related to the mechanisms that lead to the violent explosive eruptions of Bezymianny and perhaps also at other volcanoes of similar type. By integrating seismology, remote sensing, and petrological analysis of rock samples (Fig. 1) taken after the 20 December 2017 explosion, we derive a comprehensive picture of the heterogeneity immediately prior to the cataclysmic eruption.

### Tomography results: 3D velocity model and seismicity

**Figure 3:**
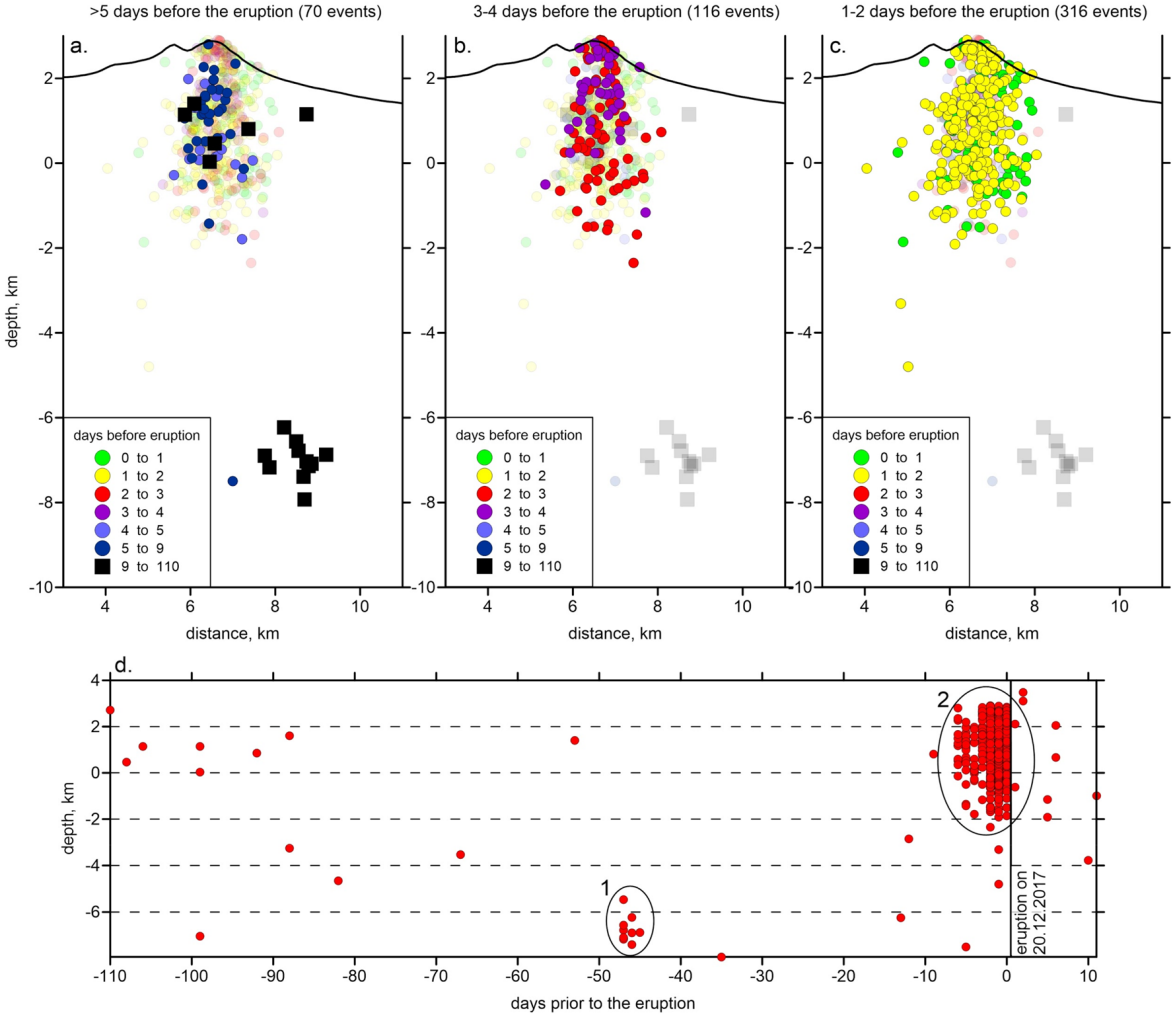
Distributions of the local seismicity at the proximity of Bezymianny recorded by the temporary network. (**a**–**c**) Locations of events in a vertical section (same as used for presenting the main results in Fig. 4) colored according to the time before the eruption. Note that during the week before the eruption, mostly shallow events were recorded. (**d**) Distribution of the focal depths versus the time prior to the eruption. Ellipses mark distinct seismicity clusters discussed in the text. The image has been produced using the Surfer Golden Software 13 (https://www.goldensoftware.com/products/surfer).

Our seismic network was deployed in August 2017 on the flanks of Bezymianny volcano (Fig. 1b) allowing us to explore the eruption preparation and culmination in high resolution. From the continuous seismograms recorded by this temporary network and the permanent seismic stations of KBGS, we identified the 523 strongest volcano-tectonic earthquakes located in the vicinity of Bezymianny. From the analysis of these event distributions, we explore the evolution of activity prior to the 20 December 2017 explosion (Fig. 3a–c). Distributions of focal depth versus time prior to the eruption shows that the background seismicity beneath Bezymianny is scarce (Fig. 3d). Besides two swarms (ellipse areas in Fig. 3d), we recorded no more than 20 volcano-tectonic events at close proximity of Bezymianny for more than 110 days of observations. Approximately 45–48 days before the eruption, seismic activity increased at depths of 6–8 km b.s.l. This swarm was followed again by a silent period of ~ 40 days, until the main cluster of shallow seismicity preceding the eruption culmination initiated 9 days before the eruption (ellipse 2 in Fig. 3d). One week before the eruption, seismicity increased markedly and was located primarily at shallow levels starting from the surface of the volcano to a depth of ~ 2 km b.s.l., whereby almost no events were detected at greater depths. Overall, the shallow seismic events arranged in a ~ 2 km wide and vertically elongated cluster located at depths between ~ 3 km a.s.l. and 2 km b.s.l. With 314 events, the bulk of the detected seismicity occurred only during the last two days prior to the eruption.

**Figure 4:**
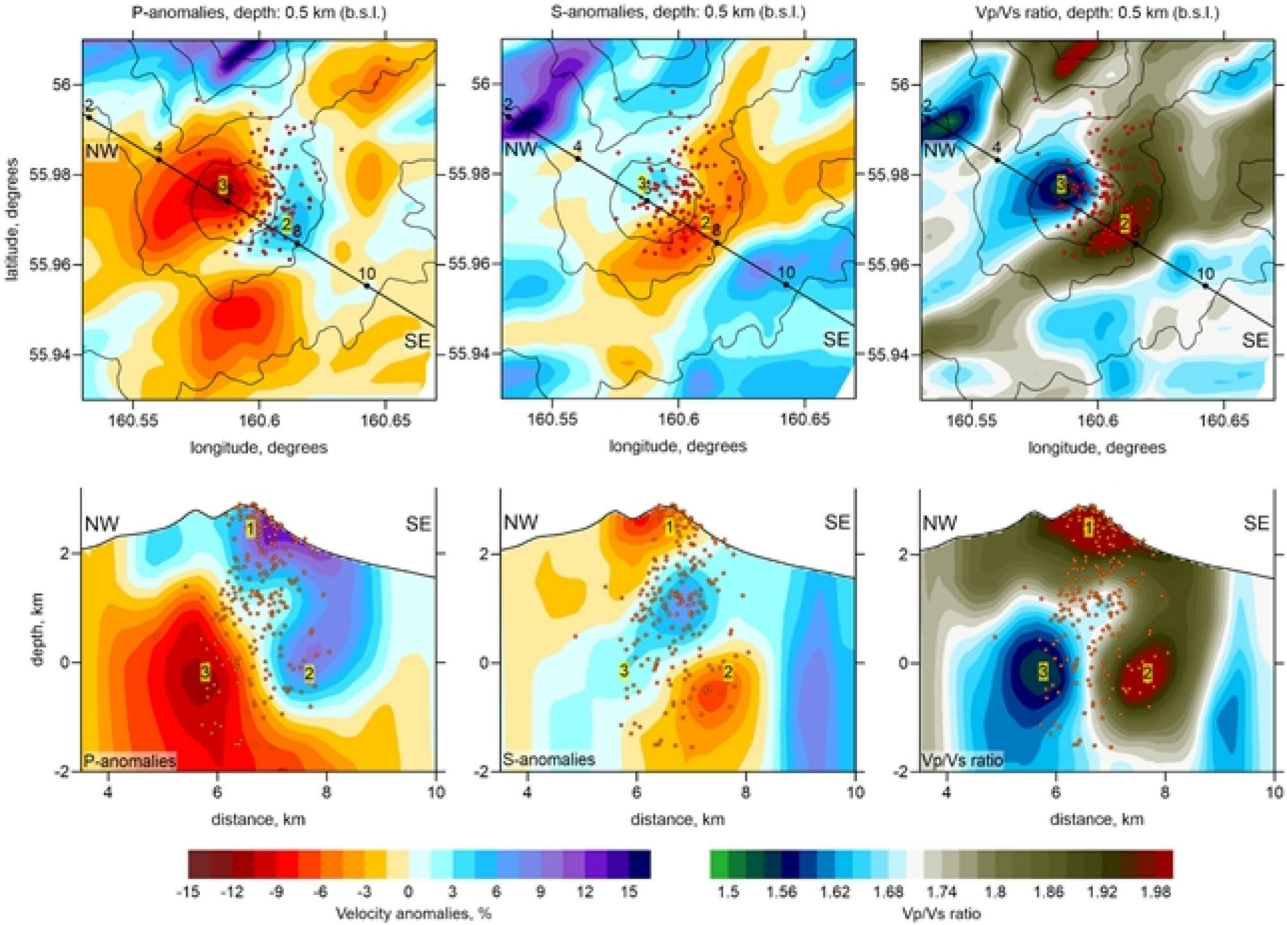
Results of tomographic inversions for the Vp and Vs anomalies and the Vp/Vs ratio in a depth level (upper row) and a vertical section (lower row). The location of the profiles is shown in the maps. Red dots indicate the locations of events at the distance of less than 0.5 km from the sections. Contour lines in the maps indicate the topography at every 500 m (http://www.marine-geo.org). Yellow numbers indicate inferred volcano surface anomaly (1), magma reservoirs (2) and gas reservoir (3). The image has been produced using the Surfer Golden Software 13 (https://www.goldensoftware.com/products/surfer).

The arrival times of the *P* and *S* waves from the recorded events located beneath Bezymianny were used to perform the tomographic inversion with the standard local earthquake tomography code LOTOS^38^ further detailed in the Methods section. The main results of tomographic inversion are presented as anomalies of the P and S wave velocities (dVp and dVs), as well as the distributions of Vp/Vs ratio shown in Fig. 4 in one horizontal and one vertical sections. More sections of this model, as well as the results of synthetic testing demonstrating the resolution limitations are presented in Supplementary materials. The generated tomographic model of the pre-eruption state of Bezymianny’s subsurface reveals strongly heterogeneous structures of pressure and shear wave velocities *(Vp, Vs)* and their ratios (*Vp/Vs)* down to ~ 2 km b.s.l. that can be interpreted as zones of fluid and magma storage or different geologic units.

### Camera and satellite radar imaging

**Figure 5:**
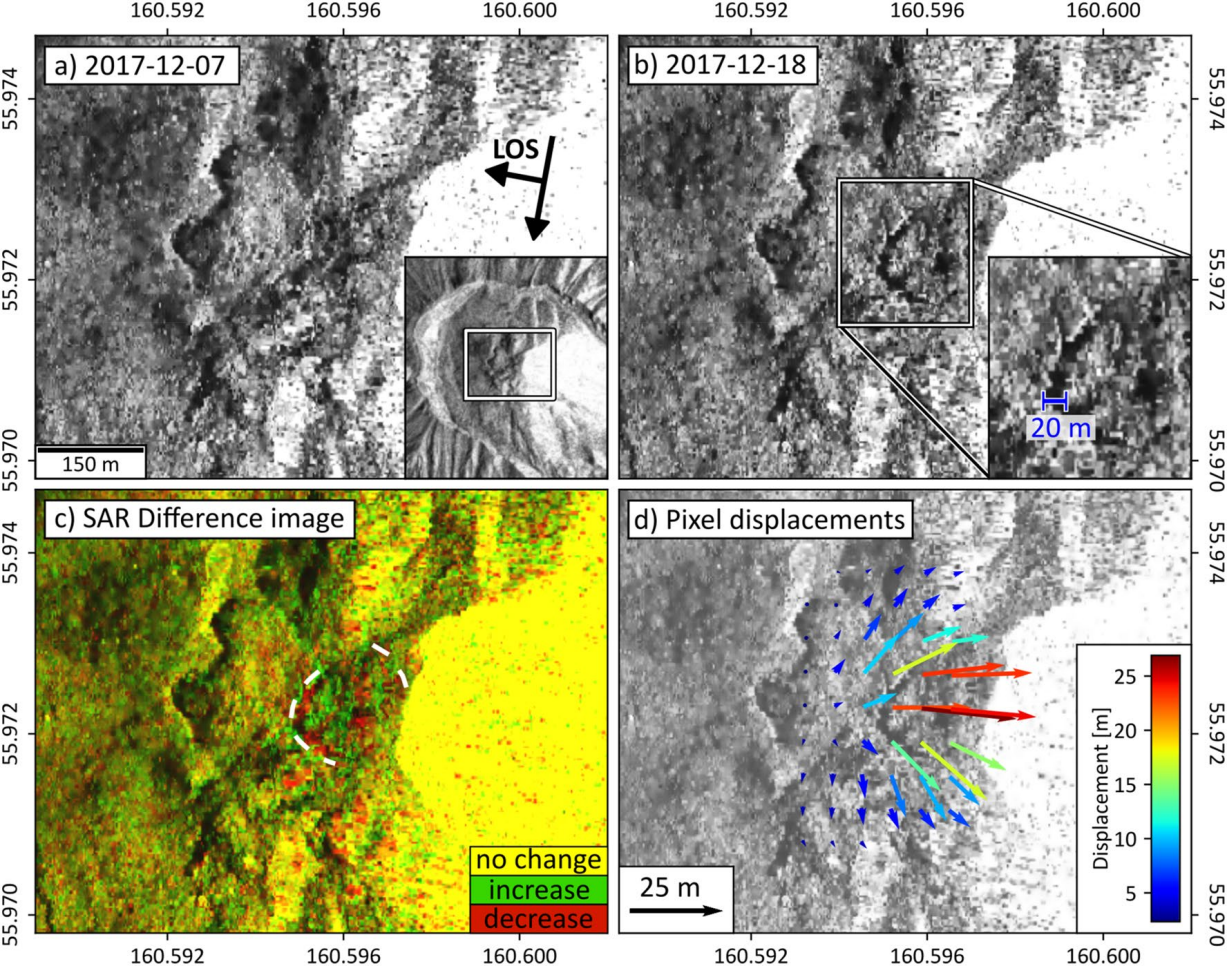
TerraSAR-X amplitude imagery of Bezymianny. (**a**) Surface reflectivity of the radar signal on 07 December 2017. Arrows refer to the azimuth and Line-of-Sight (LOS) direction of the sattelite. Inset indicates the area zoomed in for (**a**–**d**). (**b**) Reflectivity on 18 December 2017. Dark shadow regions due to opening tensile fractures (20 m wide, N–S extent, approx. 100 m) that appeared two days before the 20 December 2017 eruption. (**c**) Amplitude changes shown with red and green colored pixels. Please note the concentric pattern (white dashed lines) of newly emerged radar shadows. (**d**) Pixel offsets indicate the motion of displacement related to the opening of the shadow casting fractures and summit uplift towards LOS. Images a-c generated using GAMMA Remote Sensing software as well as Matplotlib, OpenCV libraries in Python. Image d: displacements generated with the Matlab based Particle Image Velocimetry (PIV) software PIVlab, plotted with Python. TerraSAR-X (TSX) spotlight-mode satellite images available from German Aerospace Center (DLR).

To complement our observations, we examined the images of permanently installed network cameras as well as temporarily installed (July 2017–August 2018) time lapse cameras (Reconyx XR6 UltraFire). Monitoring time lapse cameras placed by us at the eastern and southern Bezymianny flanks show steady white steaming (mostly water vapor) at Bezymianny two weeks before the eruption (Figs. 2, 5a) with fluctuations that may depend on extrinsic hydro-meteorological conditions. Images from 19 December (22:15 UTC) then reveal darkening of the steaming plume. Widespread mass wastings (rockfalls) leaving on 19 December 2017 dark brownish traces to the southeastern and eastern flank of the edifice (Fig. 2) at least five hours prior to the eruption on 20 December 2017 (03:45 UTC). The explosion images on 20 December 2017 03:45 UTC show dark ash laden convective plume rise and near surface surges covering the entire inner crater, then mantling all the time lapse cameras in dense ash 15 min later.

Bezymianny volcano is regularly studied using synthetic aperture radar (SAR) data acquired with TerraSAR-X (TSX) satellites that revealed prolonged precursory plug extrusion as well as inelastic expansion of the upper edifice flanks between 2016 and 2017^39^. Yet, the 11-day-revisit period of the individual satellite tracks prohibits short-term identification of potential precursors preceding an eruption. To minimize this temporal lag, we analyzed six amplitude images acquired between 30 November and 18 December 2017 by three different TSX tracks (Table S2, Fig. S13).

For co-registration and geocoding of the SAR images, we employed a 2-m-resolution digital elevation model^25^ (further details are described in the supplementary). Each pixel of the amplitude image depicts the intensity of the echoed radar signal, which is mainly affected by the roughness of the illuminated surface^40^. Since TSX amplitude spotlight images provide high resolution (~ 1 m/pixel), they are highly beneficial for studying surface changes at active volcanic centres^39, 41^. To determine even small-scale changes at Bezymianny’s summit region, we created amplitude difference images by merging the individual tracks’ first and last images into one RGB (Red (first image), Green (second image), Blue (empty)) image (Figs. 5, S13). Regions of increased and decreased amplitude are depicted by cyan-green and red colors, respectively, and areas of no change are illustrated by yellow colors. To further emphasize changes between the geocoded primary and secondary amplitude images of the descending track, we employed the Particle Image Velocimetry (PIV) method^42^ (for details see supplement). Comparison of two consecutive images reveals precursory deformation occurrence at the higher summit of Bezymianny volcano. In the period 07 December–18 December 2017 we identify summit expansion and a systematic shift in range direction interpreted as a doming-like uplift mechanism.

Satellite radar amplitude changes confirm the occurrence of these mass wastings on the edifice flanks seen also in time lapse cameras, some occurring already three days prior to the eruption (Fig. S13f), at a timing when the cameras were covered in fog. The satellite radar signal penetrates this bad weather and provides additional constraints. Most strikingly, however, are concentric radar amplitude changes visible at Bezymianny’s summit on 17 December 2020 (Fig. S13fh,i), whereby the extent of these concentric amplitude changes are most prominent in the image from 18 December 2020 (Fig. 5b,c). Despite the occurrence of this new large radar shadow, which is likely related to the opening of a large fracture, pixel displacements for the descending pair point only towards the viewing direction of the satellite (Fig. 5d). We interpret the latter observations as pronounced fracturing processes that are related to the pressure induced extrusion of a new precursory conduit plug, which is typically for Bezymianny prior to its eruptions^24, 39^. The temporal evolution of the amplitude changes in conjunction with enhanced degassing activity agrees well with the timing of the observed transition of deep to shallow seismicity three to four days prior to the 20 December eruption.

### Petrologic data

**Figure 6:**
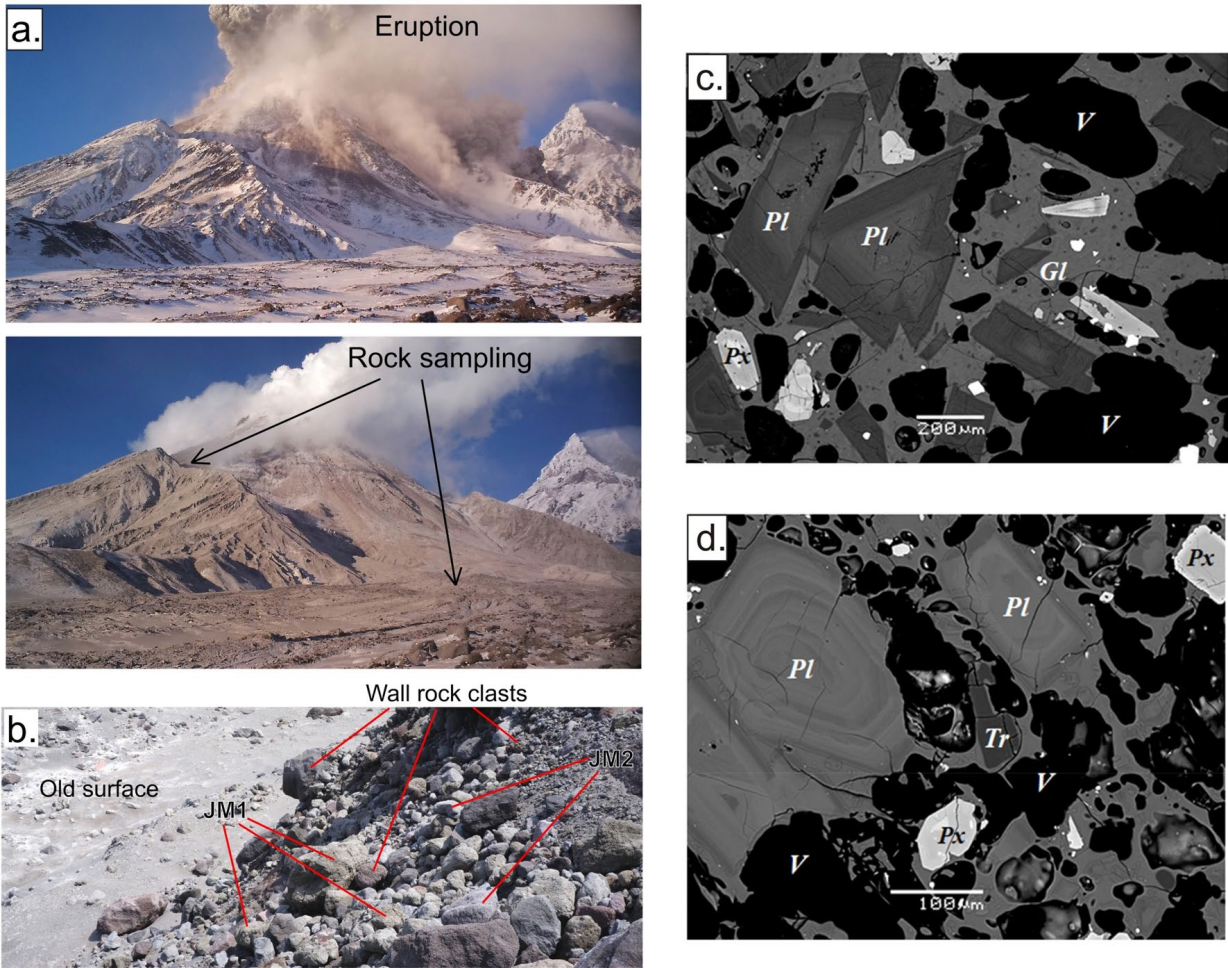
Rock sampling and petrological analysis. (**a**) Time-lapse pictures of Bezymianny during the eruption and shortly after the eruption with the indication of the rock sampling sites. (**b**) Picture of the pyroclastic flow of the recent eruption. (**c**, **d**) BSE image of two types of juvenile material: (**c**) JM1, a porous basaltic andesite with glassy groundmass. Pl—plagioclase, Px—pyroxene, Gl—glass, Tr—Tridymite, V—vapor bubbles. (**d**). JM2 porous andesite with Tridymite and glass in groundmass.

Eruptive products of the Bezymianny 20 December 2017 eruption were sampled in July 2018, synchronously with the recovery of the temporary seismic network. The pyroclastic flow deposits associated with this eruption appear to be highly heterogeneous (see Fig. 6b) and contain elements from different parts of the plumbing system. The proportion of wall rock clasts in the sampled pyroclastic flows always exceeds 10%, and in some places even 50%. This may indicate exceptionally strong gas pressurization prior to the eruption that eventually destroyed the upper edifice. This pressurization may be evidenced by the diverging amplitude pixel offsets (cf. Fig. 6d) in the outer parts of the summit crater.

We have analyzed the rock samples using the Back-Scattered Electron (BSE) imaging with the electron microscope JEOL JSM-6480 at the Geological Department of Moscow State University. We used an accelerated voltage of 20 keV, beam current of circa 10 nA and time of exposition of 80 s. Porosity and crystallinity were measured with ImageJ software. We analyzed the grayscale histogram of each BSE image and calculated integral areas for each mineral and glass. All pores in BSE images were preliminary filled with black color for more precise estimations. For each sample, we considered > 10 BSE images.

Based on the BSE imaging of the collected samples, we have found a considerable amount of juvenile material in the pyroclastic flow (tens of percent of the total volume) corresponding to rocks that were brought from crustal magma reservoirs. We can distinguish at least two types of juvenile rocks that differ by their texture and whole rock chemistry. The greenish blocks (JM1) were identified as fresh basaltic andesites (Fig. 6c) with a porosity of up to 40 vol%, 15–25% of glass, and phenocrysts of plagioclase and pyroxene. We conjecture that the JM1 samples originate from a deeper part of the shallow magmatic reservoir or magmatic conduit. Light-greyish blocks (JM2) are andesites (Fig. 6d) or basaltic andesites with phenocrysts of plagioclase and pyroxene. Their porosity varied in a wide range between 10 and 60%. Specific feature of JM2 is the presence of silica minerals (Tridymite or Cristobalite) (Fig. 6d).

## Discussion

The seismic tomography model derived in this study provides higher resolution for the upper crust beneath Bezymianny compared to previous results. In particular the tomography studies in^32, 33^, which used the data of a local network PIRE installed on Bezymianny in October-December 2009, together with datasets collected by permanent and other temporary networks, revealed a large shallow anomaly of low velocities and high *Vp/Vs* ratio beneath Bezymianny. However, they could not reveal an anomaly of low *Vp/Vs* ratio at ~ 2 km depth, which appears to be prominent in our model. This might be partly due to limited resolution of the previous studies, but may be also associated with considerable change of physical properties of rocks merely prior to the eruption in 2017.

In the resulting tomography model, we observe several zones with anti-correlating *Vp* and *Vs* wave anomalies. This may be explained by different sensitivity of these two types of waves due to various physical parameters of rocks. In many volcanic systems, for instance, the *Vp* is primarily affected by the composition of rocks and the degree of gas saturation, whereas *Vs* is strongly dependent on the presence of a liquid phase (melts or hydrothermal liquids). Therefore, integration of both *Vp* and *Vs* provides better constraints on the rock properties than using a single type of wave.

At shallow depths inside Bezymianny’s edifice, our model indicates the coexistence of a high *Vp* and a low *Vs* anomaly, resulting in a high *Vp/Vs* ratio of 1.9 (anomaly 1 in Fig. 4). While the high *Vp* can be associated with the existence of consolidated magmatic rocks, the low *Vs* may be due to the presence of considerable large amounts of liquids. This anomaly might be caused by meteoric water penetrating into the volcano edifice up to 1–2 km below the surface, as has been shown at other volcanoes^43^. Days prior to the explosion the occurrence of increased seismicity at the shallow level concurs with increased steaming and rock falls as seen in monitoring cameras, as well as updoming and fracture opening as identified in satellite radar images, implying these transients to represent a robust precursory signal.

At greater depths of ~ 3 km, located slightly off-centered beneath the southeastern flank of Bezymianny, the coincidence of high *Vp* and low *Vs* results in an anomaly of high *Vp/Vs* ratio (anomaly 2 in Fig. 4): a typical feature of many active volcanoes usually interpreted as a magma reservoir^33, 44^. In contrast, we observe the opposite pattern beneath the northwestern flank of Bezymianny (anomaly 3 in Fig. 4), where a prominent negative *Vp* anomaly (− 15%) coexists with a locally higher or neutral *Vs* anomaly. The resulting very low *Vp/Vs* ratio (1.5–1.55) indicates that the related medium behaves as a gas-filled sponge that strongly slows down the pressure waves^45^. In the literature, there are several examples of volcanoes with a high degassing level, such as Yellowstone^46^, Campi Flegrei^47^, Gorely^48^, Nevado del Ruiz^7^, and Mount Spurr^49^, for which seismic tomography revealed similar anomalies of low *Vp/Vs* ratio interpreted as gas storages. Thus, our model shows the existence of both magma and gas reservoirs that are spatially separated but located at the same depth of 2–3 km below the surface of Bezymianny.

We infer that the concert of both reservoirs controls the current eruption activity: the magma reservoir may be the source for the episodic dome growth and lava flow emplacements as observed between December 2016 and March 2017 at Bezymianny^50^, whereas the gas reservoir may lead to short but powerful explosive eruptions of Bezymianny, such as the one on 20 December 2017.

Although the vertical resolution in the lower part of our model is strongly limited, the synthetic tests demonstrate that our tomography inversion is capable of recovering vertically oriented structures down to ~ 4 km (compare Fig. S8 in Supplementary materials). Therefore, we interpret the downward extension of the anomalies with low and high values of *Vp/Vs* ratio (anomalies 3 and 2, respectively) as real and not merely associated with vertical smearing. Therefore, in Fig. 4c, we draw the vertical pathways connecting these shallow structures with deeper sources of gas and magma in mid-crustal magma reservoir. Although our data does not allow for resolving another deep-seated magma reservoir, a previous tomography study based on a regional seismic network has demonstrated a clear low-*Vs/*high-*Vp* anomaly revealing the presence of a magma source at mid crustal levels (10–15 km) beneath Bezymianny^33^.

**Figure 7:**
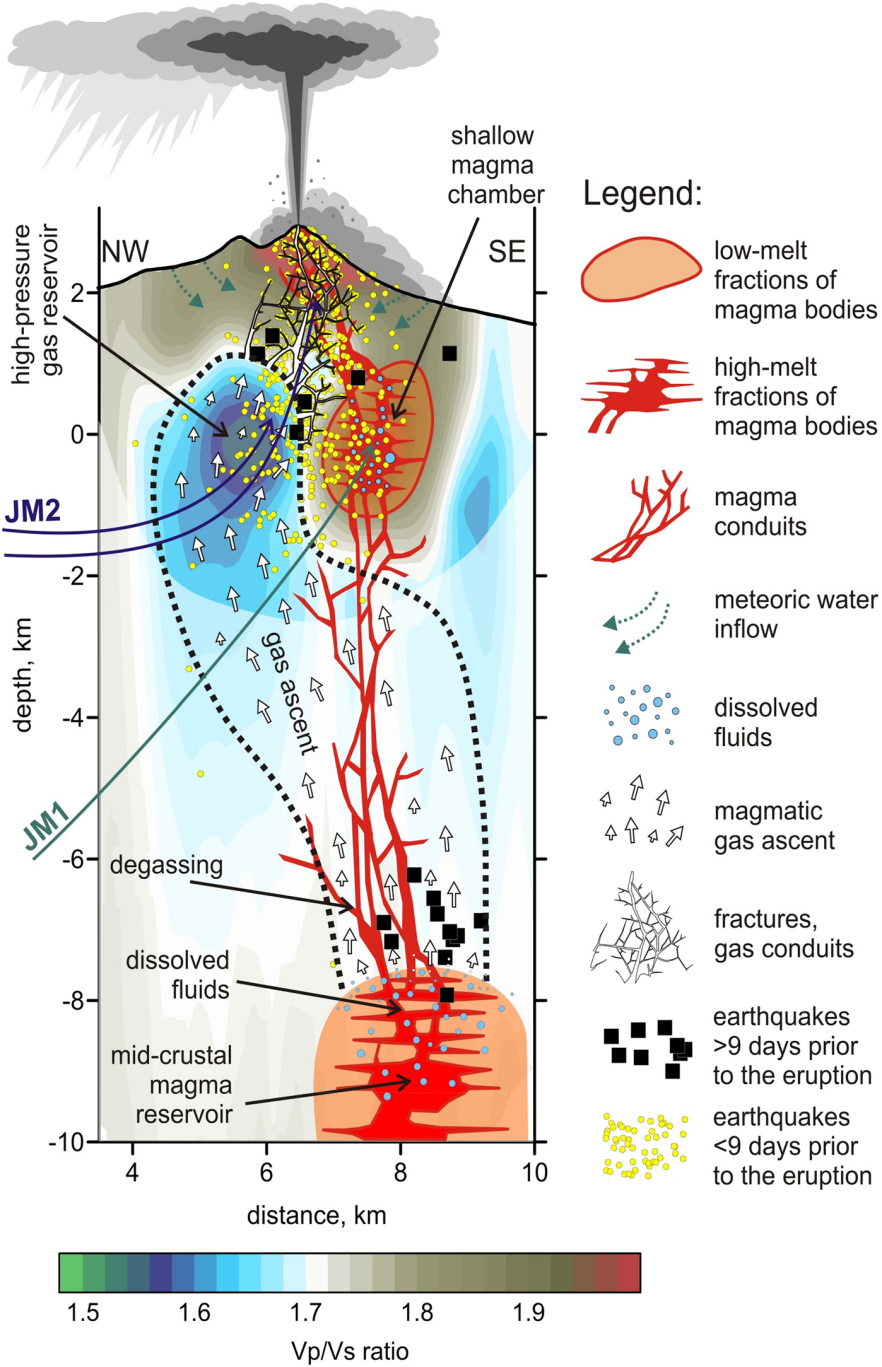
Schematic sketch of the plumbing system prior to the eruption. Background is the distribution of the *Vp/Vs* ratios along the vertical section in Fig. 4. The areas of low resolution are masked. Dotted line depicts the region of possible storage and migration of gases. Locations of the possible origins of samples JM1 and JM2 are indicated with arrows. The location of the mid-crustal magma reservoir is based on the previous larger-scale tomography study^33^.

Petrological investigations of rock samples of pyroclastic flows emplaced during the 20 December 2017 eruption have further validated our seismic deductions (Fig. 6). The pyroclastic deposits were mainly composed of wall-rocks likely ejected from the volcano edifice due to high-pressure gases. Moreover, we could also identify considerable amounts of juvenile material in the pyroclastic flows. One of the phenocryst-bearing andesite samples contained the low pressure and high-temperature-stable SiO_2_ polymorph tridymite (group JM2 in Fig. 6d), which indicates that magma was stored intermediately in the uppermost part of the shallow magmatic reservoir (Fig. 7). In addition, several JM2 samples contained the metastable SiO_2_ polymorph cristobalite, which is potentially a product of gas-magma interaction in the dome or in the conduit^50^.

Other juvenile samples (group JM1 in Fig. 6c) have basaltic-andesite composition and do not contain any silica polymorphs. We propose that they may originate from a deeper part of the shallow magma reservoir (Fig. 7). The voluminous pores in all types of the juvenile samples demonstrate the presence of gas or a gas–melt mixture that was likely the major driving force of the investigated eruption.

We hypothesize that the driving mechanisms of the 20 December 2017 eruption of Bezymianny was associated with the migration of magma as well as the ascent of large amounts of fluids into the shallow (2–3 km below surface) plumbing system, which followed a series of subsequent stages:

1. Firstly, dissolved magmatic fluids accumulated in the upper part of a potential magma reservoir below 8 km depth (Fig. 7). This reservoir is lacking earthquakes (Fig. 3), and due to the ductile rheology only provides slight mobility of volatiles^51^.
2. The second stage initiated as soon as the pressure of accumulating fluids within the upper part of the mid-crustal magmatic reservoir (stage 1) became critically high, eventually causing upward escape of fluids in combination with ongoing decompression. With decreasing pressure during ascent, the solubility of volatiles also declined, stimulating a transition from dissolved fluids into gas bubbles that was characterized by a considerable change of the volatile volume in the mid-crustal reservoir below 8 km b.s.l. This phase transition likely triggered the onset of the deepest seismic events detected 40 days prior to the eruption (Fig. 3a,d). A process also inferred from investigations on Bezymianny’s fumaroles^52^ prior to eruptions between 2007 and 2010.
3. Subsequently, the released gases from the mid-crustal magmatic reservoir migrated through a highly permeable region depicted by a wide low *Vp/Vs* anomaly (Fig. 7). In addition, this journey can be traced by the sequential occurrence of the deep cluster followed by extensive shallow seismicity reaching into the edifice of Bezymianny. Assuming a temporal gap of 40 days between the occurrence of the deep and shallow earthquakes, the gas migration velocity can be estimated to be on the order of hundred meters per day. This is a reasonable assumption for an initially highly porous and permeable medium as revealed from our petrological analysis (Fig. 6c,d), which does not contradict estimates in classical studies of gas dynamics in magmatic systems^53^. Otherwise, high porosity makes the rocks also too soft to accumulate sufficient stress released for a detectable earthquake, which may explain the lack of seismicity between depths of 8 and 2 km b.s.l. Further investigations of such temporal and spatial gaps between VT events and explosive eruptions may possibly provide the information on the material properties of the crust, which might have implications for volcano hazard forecasting (see for example the case of Lascar volcano in Chile^54^).
4. During stage four, the gas flux reached the top of the porous region between 2 and 0 km b.s.l. We propose that the anomaly of low Vp/Vs ratio (3) that derived from our tomography model represents the area of accumulation of the gases. Increasing pressure in this gas reservoir induced the rupture in the overlying rocks causing the rapid emergence of seismicity during the last days preceding the eruption. These cracks paved the way for the gas that began to reach the surface at least three days prior to the imminent eruption, as revealed by exponentially increased seismicity, enhanced degassing recorded by our time-lapse cameras (Fig. 2), and concentric amplitude changes and summit inflation determined from the TSX amplitude images and the derived pixel displacements (Fig. 5, S13). The latter indicates the gas-pressure-induced extrusion of the uppermost conduit typical for the preparation of eruptions at Bezymianny.
5. Ultimately, subsurface fractures coalesced into a single path clearing the way for gases from the shallow, high-pressure reservoir that eventually triggered the short-lived (~ ½ hour) eruption on 20 December 2017. Since magmatic gases migrated at high speeds, entrainment of abundant juvenile magma (Fig. 6 c,d) and host rock particles caused the ejection of ash to an altitude of up to ~ 15 km and emplacement of extensive pyroclastic flows.

## Conclusions

In summary, we claim that owing to the fortunate deployment of seismic stations on the flanks of the Bezymianny volcano, we obtained unprecedented insights on the behavior of the volcano feeding system prior to the volcano’s explosive eruption on 20 December 2017. The data recorded by this network were used to accurately localize the earthquakes in the volcano’s feeding system and to build a 3D tomographic model of subsurface structures beneath the volcano corresponding to a few days before the explosion. The model enabled us to identify a significant low *Vp/Vs* anomaly between depths of 0–8 km depth b.s.l. interpreted as a gas-saturated, highly permeable reservoir.

Based on the distribution of seismicity and the derived seismic tomography model as well as derivatives of satellite radar images and petrology analyses, we propose several stages of fluid migration that are responsible for the preparation of the explosive eruption. The same scenario might be valid for other explosive eruptions at Bezymianny, but also for many other explosive volcanoes in the world. Identifying large zones of low *Vp/Vs* ratios and the detection of fractures on the volcano edifice in satellite images might serve as profound forecast criteria indicating that an explosive eruption may be imminent within a few days.

## Methods

### Tomographic inversion

In August 2017, we installed a local network of portable seismic stations on the flanks of Bezymianny volcano in addition to the existing regional permanent stations of KBGS (Fig. 1b). We deployed ten seismic stations, but by December 2017, four stations were destroyed due to harsh natural conditions and because of bear attacks. Yet, the remaining six stations, together with the surrounding permanent KBGS stations, contributed to the localization of seismic events beneath Bezymianny in unprecedented quality and provided sufficient data coverage for the tomographic inversion.

The arrival times of the *P* and *S* waves from the 523 local events in the vicinity of Bezymianny were used to perform the tomographic inversion. We used a standard local earthquake tomography code LOTOS^38^, which performs the iterative simultaneous inversions for the *P* and *S*-wave velocity models and source locations. All the models presented in the main paper and in the supplementary materials can be easily reproduced using the full version of the LOTOS code with all data folders corresponding to the Bezymianny experiment, which can be found at www.ivan-art.com/science/bezym_lotos.zip.

The procedure starts with the absolute source location using the grid-search method. In the version used in this study, at this step, the approximate travel times were computed along the straight lines, which make the calculations much faster. In the next step, however, the sources are recalculated using the 3D ray tracing based on the bending method^55^. The 3D velocity model is defined in nodes distributed in the study area according to the ray coverage. In the areas with sufficient coverage, the horizontal spacing was uniform and equal to 0.4 km. In the vertical direction, the node spacing depended on the ray sampling, but could not be smaller than 0.4 km. To make our results grid independent, we performed the inversions in four different grids having different basic orientations and then average the calculated models.

The inversion of the first-derivative matrix was performed using the LSQR method^56, 57^. We performed the simultaneous inversion for the 3D distributions of the *P* and *S*-wave velocity anomalies, for source parameters (coordinates and origin times) and station corrections. To stabilize the solutions for the velocity models, we used two types of regularization: amplitude damping and flattening. Note that the values of *Vp/Vs* ratio presented as the main results of this study were computed by division of derived *Vp* and *Vs*. The robustness of this method is demonstrated by synthetic tests shown in Supplementary.

The iterative tomography cycle included the steps of source locations in the updated 3D velocity models, matrix calculations and inversions. The total number of iterations in our case was five. The values of most controlling parameters were tuned using the synthetic modeling.

To find the best reference model, we performed a series of runs of the full tomographic procedure with gradually updating starting velocity models. After each run, we determined the average *P* and *S* wave velocities in a set of horizontal levels, which were then used to define the reference model in the next run. As a result, after several runs of this procedure, we obtained a velocity model that provided a good balance between positive and negative velocity anomalies. The final 1D reference model is presented in Table 1.

**Table 1:** The reference velocity model used for the inversion of experimental data.

Depth (km)	Vp (km/s)	Vs (km/s)
− 5	3.6	2.02
− 1	3.99	2.24
1	4.14	2.32
3	4.35	2.44
5	4.65	2.61
7	4.92	2.76
30	7.8	4.38

## Supplementary information

Supplementary Information
