# Supplementary material for: Anatomy of the Bezymianny volcano merely before an explosive eruption on 20.12.2017
Source: Sci Rep. 2021 Jan 19;11:1758. doi: 10.1038/s41598-021-81498-9 (PMC7815830; doi:10.1038/s41598-021-81498-9)
Supplement: Supplementary file 1 — Supplementary Information [file 41598_2021_81498_MOESM1_ESM.pdf]

Supplementary materials for the paper "Anatomy of the Bezymianny volcano merely before an explosive eruption on 20.12.2017"

by Ivan Koulakov, Pavel Plechov, Thomas R. Walter, René Mania, Sergey Z. Smirnov, Ilyas Abkadyrov, Andrey Jakovlev, Vesta Davydova, Sergey Senyukov, Natalia Bushenkova, Angelika Novgorodova, Tatyana Stupina, and Svetlana Ya. Droznina

In this supplementary file, we present some technical details that support the conclusions presented in the main paper.

Seismic data description:

The Klyuchevskoy Group of Volcanoes (KGV), where Bezymianny volcano is located, includes three presently active volcanoes and a dozen dormant and extinct volcanoes. In the KGV, there are 17 permanent stations of the Kamchatkan Branch of Geophysical Survey of Russian Academy of Sciences (KBGS) that transfer the data in real time in continuous regime to the central office to Petropavlovsk-Kamchatsky. These data are operatively processed and used to localize seismic events in KVG[1S]. This network provides continuous information on seismicity for decades, which is an important material for studying the general state of the volcanoes within the KGV. However, the distribution of the stations is rather sparse and cannot ensure accurate locations of sources in some areas. Furthermore, standard location techniques used by KBGS to create the earthquake catalogues are based on the 1D velocity models that may cause considerable bias in respect to the true locations. Implementing realistic 3D velocity models makes possible to reduce the error of source locations.

To improve the locations of seismicity beneath Bezymianny, in summer 2017, we have deployed a temporary seismic network on the Bezymianny volcano. Initially, the network consisted of ten stations of which six were equipped with the GURALP-6T sensors and Data-Cube data loggers. The remaining four stations used the Russia-manufactured broadband sensor CMG-4111 and the Baikal data logger. Unfortunately, two stations were destroyed by bears and two stations had technical problems making the recorded seismograms useless. Thus, in December 2017, when an eruption occurred, only six portable stations provided high-quality records. Note that some of the stations were located at a distance of less than one kilometer from the explosion epicenter and we were inquired if they might be damaged or

lost due to powerful pyroclastic flows. Fortunately, all stations were successfully recovered after one year of autonomous operation in July 2018.

In this study, we used only relatively strong events that were identified with the permanent stations and reported in the KBGS catalogues. We took into account the preliminary determinations of these events and picked the arrival times at temporary stations. For picking, we used a software DIMAS[2S], which is specially adapted to analyze the volcano seismicity and used as a routine tool at KBGS. A screenshot with an example of an event picking using the DIMAS software is presented in Fig. S2. In total, the number of events was 523, most of them occurred during one week before the eruption. As we selected the strongest events, the arrivals of seismic wave arrivals were clearly identified, and the accuracy was estimated as high (0.02-0.05 s). We manually picked 9967 arrival times at the permanent and temporary stations including 4749 P- and 5218 S-wave picks. The average ratio of picks per event was ~19. In Fig. S3, we show the distributions of the ray paths of the P and S waves in horizontal and in two orthogonal vertical projections. It can be seen that the densest ray coverage is observed above the depth of 1 km b.s.l. At deeper levels, the ray distribution is much poorer.

Synthetic modeling

Prior to presenting the results of experimental data inversion, we show some results of synthetic modeling, which is an essential stage to assess the resolution limitations and to estimate the values of optimal controlling parameters. The geometry of the source-receiver pairs is taken from the experimental dataset; the source locations correspond to the solution in the final 3D velocity model. The synthetic model is defined as a superposition of the absolute 1D velocity and 3D anomalies. The synthetic travel times are computed by the 3D ray tracing using the bending method and perturbed with a random noise. In our case, we defined the mean deviation of noise equal 0.03 s, which provided similar variance reduction as in the case of experimental data inversion. After computing the synthetic data, we “forgot” the information about the coordinates and origin times of sources and started the recovering procedure with determination of preliminary source locations in the starting 1D velocity model. As will be shown below, at this stage, the sources are strongly mislocated, but after

several iterations of the tomographic inversions, the errors of source relocations were considerably reduced. When performing the recovery of the synthetic model, we used the same calculation steps and same controlling parameters as those used to calculate the preferred tomography model based on the experimental data.

In Fig. S4, we present a series of spike tests with one, two and four anomalies having the lateral sizes of 1 km and amplitudes of *dVp* and *dVs* ±9%. In these models, the *P* and *S* wave velocity anomalies had the opposite signs to enable strong variations of *Vp/Vs* ratio. The anomalies in the considered models remained unchanged with depth. The synthetic data were perturbed by noise with the mean magnitudes of 0.03 s. After performing five iterations of tomographic inversion procedure, we achieved relatively small variance reduction at an order of 15-20%, which was much lower than in the case of experimental data inversion (40-50%). However, despite of the low signal/noise level in this series of the models, we robustly recovered all the target anomalies at correct locations, though with considerably lower amplitudes than was defined in the original model. Note also that the anomaly size of 1 km is smaller than the structures we recovered after the experimental data inversion.

In Fig. S5, we show a comparison of the Vp/Vs ratio distributions derived from the recovery of two checkerboard models having the opposite (plots a and b) and equal (plots c and d) values of the P and S wave velocity anomalies. In both cases, we defined alternated squared anomalies of 1.5 km size with the amplitudes of ±9% separated by an interval of 0.5 km with zero anomalies. In these cases, the anomalies remained unchanged with depth. In the first case, the opposite values of *dVp* and *dVs* enabled strong variations of the synthetic *Vp/Vs* values, whereas equal values of dVp and dVs in the second model led to a constant value of the Vp/Vs ratio. In the first case (Figure S5 a and b), the recovery provided a robust image of the Vp/Vs ratio variations in the central part of the study area. In the second case (Figure S5 c and d), instead of an originally constant value, the inversion result showed some minor variations of Vp/Vs ratio, which was possibly caused by slightly non-uniform resolutions of recovery of the P and S wave velocity models.

In the next test shown in Fig. S6, we exploit the capacity of the inversion to resolve anomalies merely in the deep part of the model. In this case, the anomalies are defined at depths below 0.5 km b.s.l.; above this level, the synthetic anomalies are zero. In this case, we considered larger anomalies (2 km of anomaly and 0.5 km of empty spacing). The amplitude of the initial anomalies was again ±9%. The recovery result for this model shows that all the

anomalies are restored at correct locations, but in this case, the amplitudes of anomalies appear to be much lower than those in the original model. This fact should be taken into account when interpreting the main tomography results.

To explore the vertical resolution, we have performed a series of tests shown in Figs. S7 and S8. In all cases, the synthetic models were set along three different vertical sections. In the section plain, the anomalies were defined as a checkerboard model; across the section, the anomalies remained unchanged within a band of 2 km width. In Fig. S7, the size of the anomalies was 1.5 km and the amplitude was ±9%. In the recovery results, we see that within the volcano edifice, the anomalies are correctly recovered; however, outside the volcano and at depths below 1 km b.s.l., the anomalies are completely smeared. In Fig. S8, we explore the vertical resolution with larger anomalies having only one transition at the depth interval from 0.5 km a.s.l. to 1 km b.s.l. In horizontal direction, we defined an empty interval between anomalies of 1 km width. We see that in this case, most anomalies are restored correctly. Based on these tests, we have defined an area for presenting the results (Fig. 4 of the main paper), for which we can ensure sufficient resolution, with the lower limit at a depth of 2 km b.s.l.

In Fig. S9, we present an example of source locations based on a synthetic model shown in the middle column in Fig. S8. It can be seen that at the initial step of source locations in the starting 1D velocity model, the coordinates of the events are strongly biased in respect to their true locations. The mean error of source locations in this case is 0.45 km. After five iterations of the inversion procedure, the relocations of sources in the recovered velocity model leads to more than four times smaller mean error (0.11 km). This example shows that using the 1D models for source location in highly heterogeneous areas may cause considerable bias of sources. This should be taken into account when using standard location tools for producing earthquake catalogues.

Tomographic inversion of experimental data

To derive the three-dimensional distributions of the *P* and *S* wave velocities, we performed five iterations of tomographic inversions. Actually, the number of iterations has a similar effect as the amplitude damping and smoothing. Approximately same solutions can be obtained for cases of many iterations with strong damping and fewer iterations with weaker

damping. Therefore, in our workflow, we usually fix the number of iterations (5 in the present case), and then tune the damping values to enable the best recovery of synthetic models. The reduction of the residuals in the *L1* norm during the inversion procedure in five iterations is reported in Table S1. It can be seen that the *P* wave residuals have reduced from 0.162 s to 0.094 s (42%) and the *S* wave residuals evolved from 0.261 s to 0.133 s (48%). Such considerable reduction is rarely obtained for such types of local networks. Actually, short source-receiver distances prevent accumulation of large residuals, which remain compatible with noise. In our case, such high reduction indicates that the signal to noise ratio is high, which has two reasons: high quality of the picking data and strongly heterogeneous velocity structures in the study area that were successfully recovered by seismic tomography.

Table S1. Values of the P and S wave residuals in the L1 norm and their reduction during the iterative procedure

Iteration	mean dtp, s	reduction dtp, %	mean dts, s	reduction dts, %
1	0.162	0	0.261	0
2	0.102	37.10	0.146	44.01
3	0.097	40.06	0.139	46.73
4	0.095	41.39	0.136	47.90
5	0.094	42.15	0.133	48.89

The results of inversion for the anomalies of *Vp* and *Vs*, as well as for the *Vp/Vs* ratio are shown in the main paper in one horizontal and one vertical sections (Figure 4). Here, we show additional horizontal sections in Fig. S10 and three vertical sections in Fig. S11. Let us remember that the *Vp/Vs* ratio is obtained from the division of absolute values of *Vp* over *Vs* independently calculated during the tomographic inversion. The robustness of such a method was carefully tested by synthetic modeling.

In the horizontal section at -1 km asl (Fig. 10 left column), the higher *Vp* coexists with a prominent negative *Vs* anomaly, which results at high *Vp/Vs* ratio reaching 1.9. In the previous study[3S], the same shallow anomaly with high Vp/Vs ratio was detected beneath Bezymianny. The high *Vp* coinciding with the location of the volcano edifice can be associated with the consolidated magmatic rocks that form the volcano edifice. On the other hand, low *Vs* and high *Vp/Vs* ratio can be associated with the presence of a big amount of fluids, for example, with meteoric water penetrating into the volcano edifice down to 1-2 km[4S]. Similar features were previously observed in many different volcanoes, such as: Nevado del Ruiz[5S], Redoubt[6S] and others.

At 1 km b.s.l., the *P*-wave velocity has the opposite value compared to the shallower section. Very low *Vp* may indicate that the medium here behaves as a sponge saturated with gas that strongly attenuates the pressure waves[7S]. The *S*-wave model at 1 km b.s.l. right beneath the volcano represents a locally higher velocity anomaly surrounded by low velocities. The *Vp/Vs* ratio at this depth appears to be low that may also be an indicator for gas contamination.

It is interesting that the negative *S*-wave anomalies at 1 km depth form a structure elongated in the SW-NE direction. It is possible that this structure is related to a regional fault that crosses the entire KGV and is considered as a structure attracting the presently active volcanoes. It is known that many volcanoes tend to be associated with tectonic faults as they produce mechanically weakened media, which are favorable for migration of fluids and melts.

In Fig. S12, we present the results in three vertical sections for the absolute velocities of the *P* and *S* waves. We can see that the summit area the distributions of velocity are highly heterogeneous and sometimes cause the inversion of velocities when at deeper the velocity appears to be lower than in the shallower layers. In this case, using a 1D model for localizing seismic events would obviously cause considerable errors.

Time lapse camera recording

In order to investigate surface changes associated with volcanic activity at Bezymianny, we designed a network of time lapse cameras. In total 5 cameras were installed, one was destroyed by the explosion, one was blurred by internal ice, and three showed good quality imagery. These three cameras (C1, C2 and C5) were installed C1: N55.94878, E160.63167,

H=1592 m, C2: N55.96285, E160.65021, H=1684 m, and at C5: N55.95334, E160.58594, H=2120 m. We used Reconyx UltraFire trail cameras, which were set to take an image every 15 minutes at a resolution of 2304 × 1296 pixels. The 5 mm lens has a full frame equivalent focal length of 37 mm, which is why we placed the cameras at a distance of 2-3 km from the volcano summit. We used internal lithium batteries to power the cameras, and the internal clock was controlled by synchronization to a GNSS station before the installation and controlled again at the end of the experiment to account for possible time drift. We placed all cameras directly onto a big solid boulder, where we drilled and glued screws for fixing the camera mounts and steel bear protection housings. The camera data allows identifying white steam clouds, the occurrence of rock falls and mass wasting, and occurrence and duration of eruptions. The steaming of the volcano is also affected by hydrometerological conditions, such as temperature and air pressure, and therefore needs to be considered with care.

Synthetic Aperture Radar (SAR) processing

SAR satellites illuminate the Earth's ground with radio waves and record the echoed phase, frequency, and amplitude information. The latter is modified by the roughness and dielectric properties of the investigated surface that results in images depicted by darker and brighter pixels induced by rougher and smoother surfaces respectively[8S]. Due to its daytime independence and the ability to penetrate ash clouds, amplitude images had been highly beneficial to investigate hazardous volcanic processes such as dome growth[9S,10S] and to mitigate associated hazards[11S]. Here, we take advantage of changes in high-resolution TerraSAR-X (TSX) spotlight-mode amplitude images acquired from two ascending (ASC) tracks and one descending (DSC) track (Table S2, Fig. S13), to additionally investigate surface changes prior to the 20 December 2017 eruption of Bezymianny.

For co-registration and image extraction of subsequent amplitude scenes of the individual tracks, we used the Gamma remote sensing software[12S] and a 2-m-resolution digital elevation model (DEM) [13S]. The images are vividly characterized by radar shadows, foreshortening, as well as layover effects[10S]. To reduce speckle noise while preserving edges around darker and brighter amplitude regions, we applied the bilateral filter from the OpenCV library in Python3[14S]. We chose a kernel size of 7 pixels, a smaller spatial weight of 25 (euclidean distance) to reduce salt and pepper noise but with less effect of pixels further away, and a spectral weight of 75 to improve edges in the images. Subsequently, we enhanced the images' contrast with the createCLAHE (Contrast Limited Adaptive Histogram

Equalization) module by setting the tile size to 16x16 pixels. To avoid noise amplification, we used a clipping limit of 2 before histogram equalisation is applied. Eventually, we used the OpenCV merge module to create RGB images, where the individual first and secondary images represent the red and green channels, respectively. The resulting difference images (Fig. S13c,f,i) highlight regions of amplitude increase (cyan), decrease (red), and no change (yellow), similar to the approach described in[15S].

To further enhance the visualisation of changes and to quantify the amount of displacement between the georeferenced primary and secondary amplitude images of the descending track, we employed the Particle Image Velocimetry (PIV) software PIVlab16S, 17S. The original amplitude images were pre-filtered by using the Wiener denoise filter with a kernel size of 2 pixels. Subsequent cross-correlation was done through a fast Fourier transformation by using four interrogation windows with successively smaller sizes (80, 64, 48,40 pixels), while the step size each time was half that of the window size. The location of sub-pixel displacements were estimated with a 2x3 point fit Gaussian function. Eventually, the detected pixel displacements were smoothed in order to remove the amount of tracked noise.

In particular, the difference image of track 64 reveals that until 11 December 2017, no significant changes occurred at the summit of Bezymianny (Fig. S13c). First considerable changes are discernible in the track 155 difference image (6-17 December 2017) and are characterised by marked amplitude decreases along the eastern flank of the edifice (Fig. S13f), which we interpret as the result of the sliding of rock avalanches. Moreover, the latter image depicts slight concentric amplitude changes at the summit, which may be related to surface uplift related to enhanced pressurization induced from the conduit that initiated between 11-17 December 2017. Strikingly, the track 11 difference image (7-18 December 2017) uncovers also concentric radar shadows at the summit that are much more prominent to the west than to the east of the summit (Fig. 13i). The concentric arrangement as well as area-wide reflectivity increase at the summit may indicate reinforced fracturing likely caused by the rise of magmatic fluids pushing a potential upper conduit plug as was described in previous studies[10S]. The fact that we do not observe expansion of Bezymianny's cone as observed prior to the December 2016 and March 2017 eruptions[10S], indicates that the preparation stage of the imminent December 2017 eruption was mostly driven by magmatic fluids other than magma.

Table S2. Key parameters of ascending (ASC) and descending (DSC) TSX acquisitions.						
**Pass**	**Track no.**	**Az. [°]***	**Inc. [°]****	**Range px. Spacing [m]**	**Azimuth pixel spacing [m]**	**Pixel size [m]*****
ASC	64	351.4	48.7	0.91	1.21	2.1
ASC	155	349.6	39.7	0.91	1.26	2.1
DSC	11	190.6	38.7	0.91	1.26	2.1
* Azimuth (flight direction) angle; ** Incidence angle; *** Size in x and y direction of the geocoded amplitude images						

References for the supplementary

1S. Chebrov, V.N. *et al.* The development of the system of integrated instrumental monitoring of volcanoes in the Far East Region. *Seismic Instruments* **49**, 3, 254-264 (2013).

2S. Droznin, D V., & Droznina, S.Y. Interactive DIMAS program for processing seismic signals. *Seismic Instruments* **47**, 215–224 (2011).

3S. Ivanov, I. *et al.* Magma sources beneath the Klyuchevskoy and Bezymianny volcanoes inferred from local earthquake seismic tomography. *J. Volcanol. Geotherm. Res.* **323**, 1, 62-71 (2016).

4S. Hurwitz, S., Kipp, K.L., Ingebritsen, S.E. & Reid, M.E. Groundwater flow, heat transport, and water table position within volcanic edifices: Implications for volcanic processes in the Cascade Range. *J. Geophys. Res.* **108**(B12), 2557 (2003).

5S. Vargas, C.A. *et al*. Breathing of the Nevado del Ruiz volcano reservoir, Colombia, inferred from repeated seismic tomography. *Science Report* **7**, 46094 (2017).

6S. Kasatkina, E., Koulakov, I., West, M. & Izbekov, P. Seismic structure changes beneath Redoubt Volcano during the 2009 eruption inferred from local earthquake tomography. *J. Geophys. Res. Solid Earth* **119**, 4938–4954 (2014).

7S. Takei, Y. Effect of pore geometry on VP /VS: From equilibrium geometry to crack. *J. Geophys. Res.*, **107**(B2), 2043 (2002).

8S. Massonnet, D. & Feigl, K.L. Radar interferometry and its application to changes in the Earth's surface. *Reviews of Geophysics* **36**(4), 441-500, doi: 10.1029/97RG03139 (1998).

9S. Wang, T., Poland, M.P. & Lu, Z. Dome growth at Mount Cleveland, Aleutian Arc, quantified by time series TerraSAR-X imagery. *Geophys. Res. Lett.* **42**(10) 614-621, https://doi.org/10.1002/2015GL066784 (2015).

10S. Mania, R., Walter, T.R., Belousova, M., Belousov, A. & Senyukov, S.L. Deformations and Morphology Changes Associated with the 2016–2017 Eruption Sequence at Bezymianny Volcano, Kamchatka. *Remote Sens.* **11**, 1278 (2019).

11S. Pallister, J.S. *et al.* Merapi 2010 eruption-chronology and extrusion rates monitored with satellite radar and used in eruption forecasting. *J. Volcanol. Geotherm. Res.* **261**, 144–152 (2013).

12S. Werner, C., Wegmüller, U., Strozzi, T. & Wiesmann, A. GAMMA SAR and interferometric processing software. Proceedings of the ERS-ENVISAT Symposium, Gothenburg, Sweden, 16–20 October (2000).

13S. Shevchenko, A. *et al.* The rebirth and evolution of Bezymianny volcano, Kamchatka after the 1956 sector collapse. *Nature Communications*, Paper accepted for publication, COMMSENV-20-0077B (2020).

14S. Bradski, G. The OpenCV Library. *Dr. Dobb's J. Software Tools* **25**, 120-125 (2000).

15S. Wadge, G. *et al.* Rapid topographic change measured by high-resolution satellite radar at Soufrière Hills Volcano, Montserrat, 2008–2010. *J. Volcanol. Geotherm. Res.* **199**, 142-152 (2011).

16S. Thielicke, William, and Eize J. Stamhuis. "PIVlab – Towards User-Friendly, Affordable and Accurate Digital Particle Image Velocimetry in MATLAB." Journal of Open Research Software, vol. 2, Ubiquity Press, Ltd., doi:10.5334/jors.bl (2014).

17S. William Thielicke (2020). PIVlab - particle image velocimetry (PIV) tool with GUI (https://github.com/Shrediquette/PIVlab/releases/tag/v2.36.5), GitHub. (October 29, 2020).

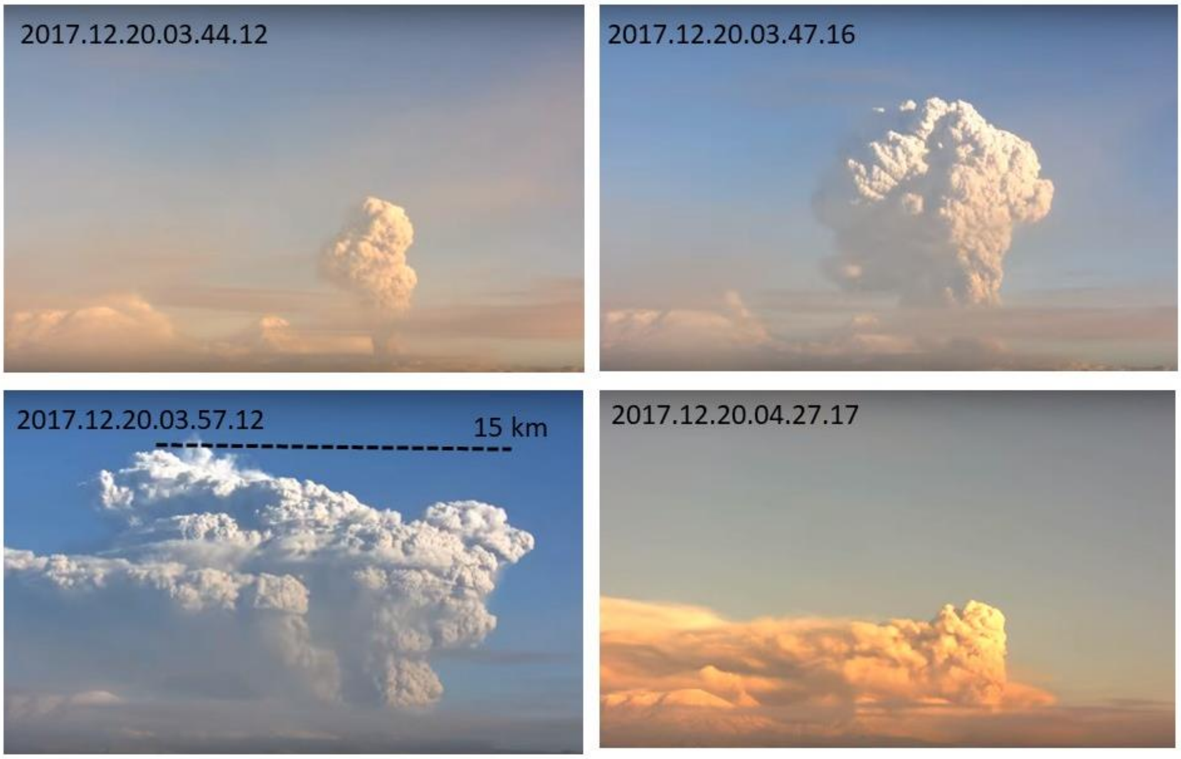

Figure S1. Images from the KBGS stream camera that recorded the explosive eruption of the Bezymianny volcano on December 20, 2017. Note that the ash plume reached the altitude of ~15 km for less than 15 minutes after the beginning of the eruption.

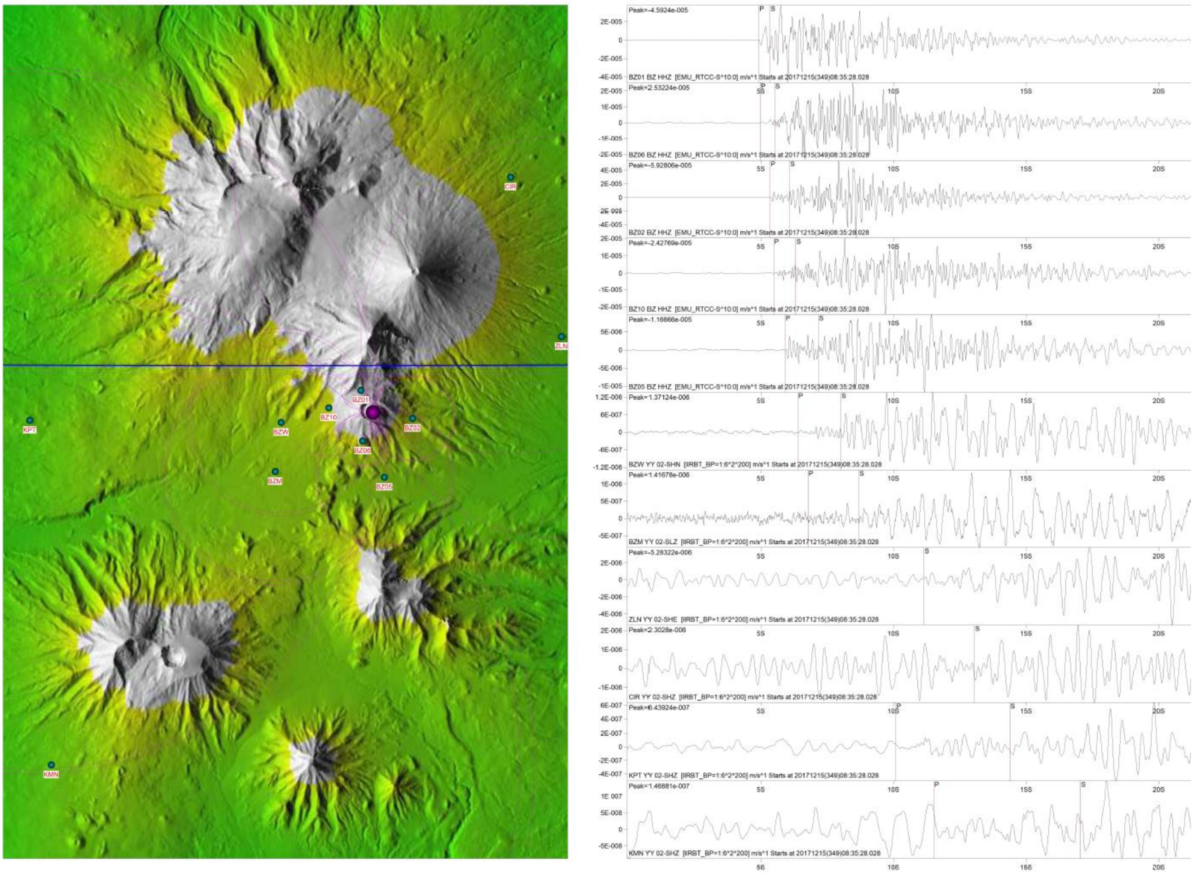

Figure S2. Example of an event picking and preliminary source location (violet dot) based on the data of the permanent and temporary networks (green dots) using the DIMAS software [2S]. Note that here we plot only the vertical components, whereas the S-waves were mostly picked on the horizontal channels.

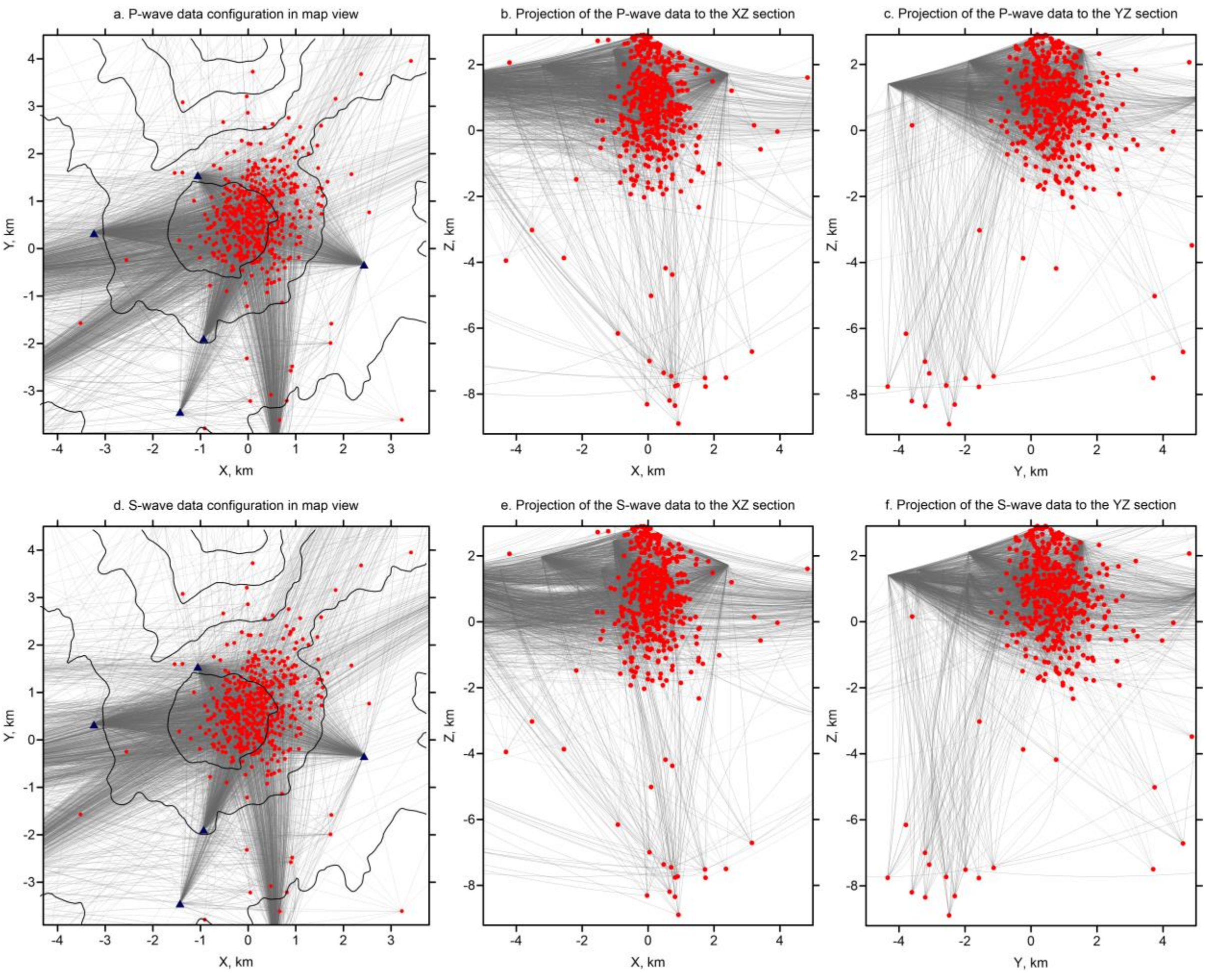

Figure S3. Ray path of the P and S waves (grey lines in the upper and lower rows, respectively) from local events (red dots) used for tomographic inversion which are presented in one horizontal and two orthogonal vertical projections. Blue triangles are the seismic stations, and black contour lines depict relief with the interval of 500 m (http://www.marine-geo.org). The image has been produced using the Surfer Golden Software 13 (https://www.goldensoftware.com/products/surfer).

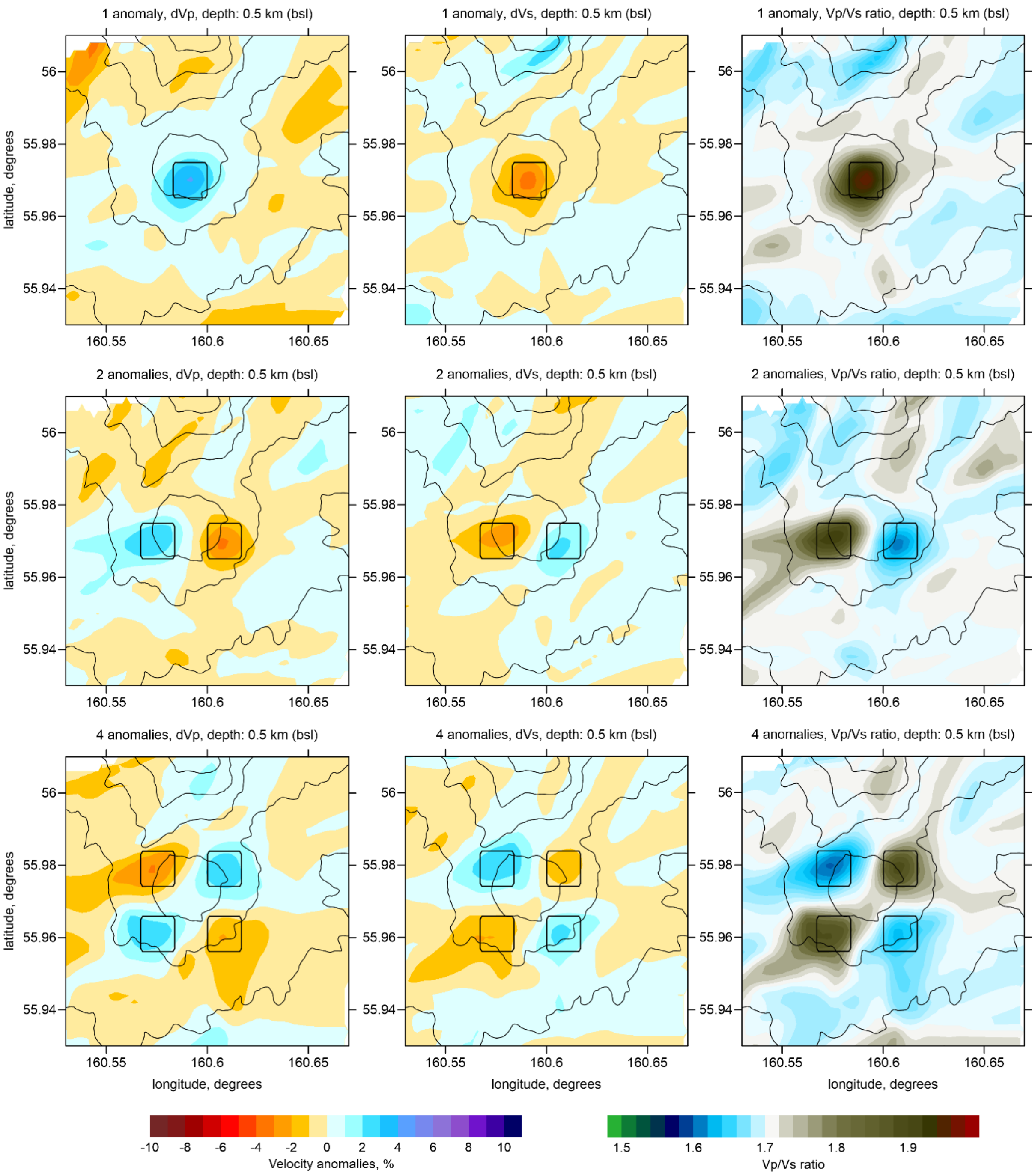

Figure S4. Three synthetic tests with one, two and four anomalies having the lateral sizes of 1 km and amplitudes ±9% (contour lines depict the shape of synthetic anomalies). With depth, the anomalies remain unchanged. The contour lines of topography are presented with an interval of 500 m (http://www.marine-geo.org). The image has been produced using the Surfer Golden Software 13 (https://www.goldensoftware.com/products/surfer).

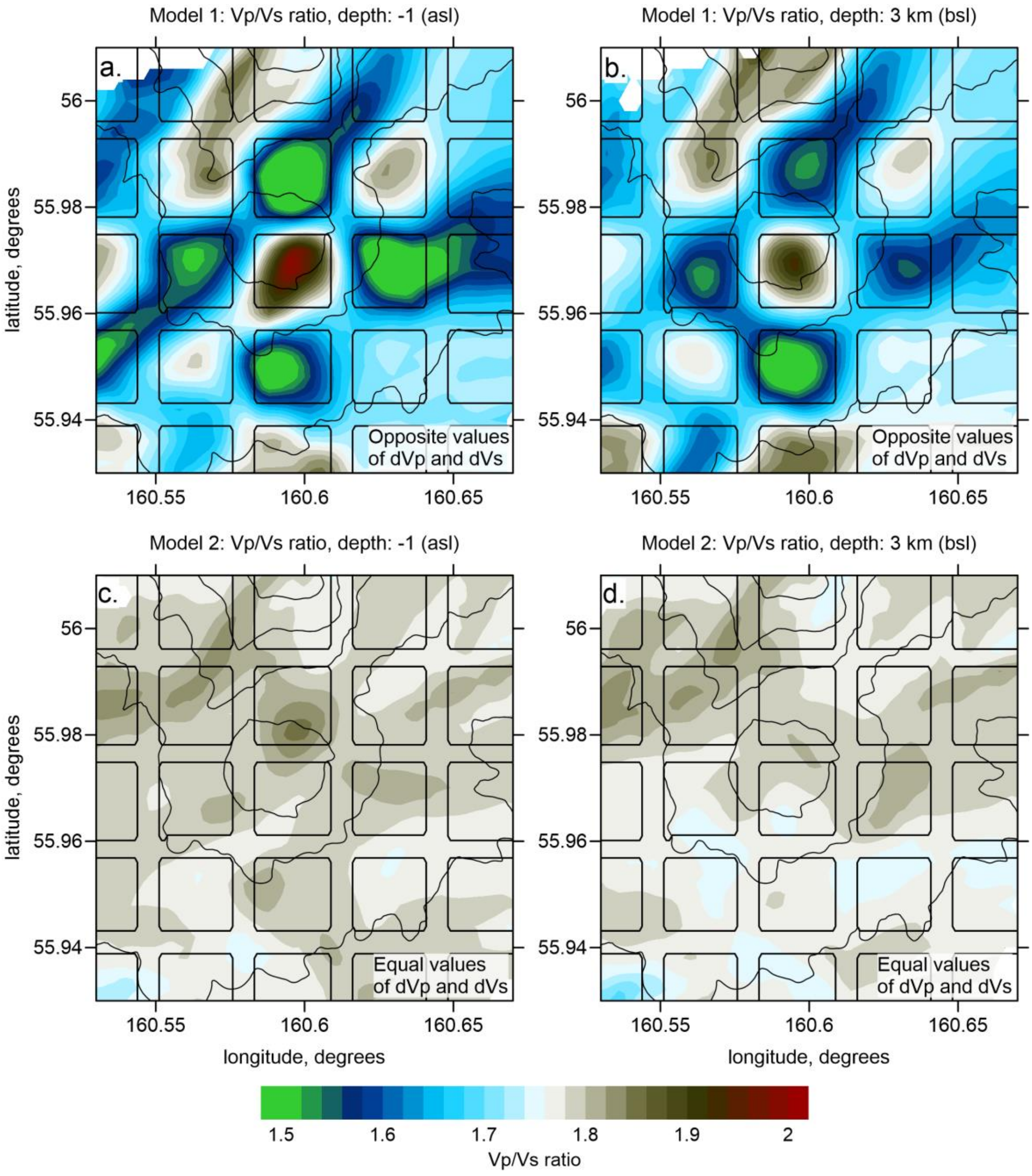

Figure S5. The distributions of the recovered Vp/Vs ratios in two checkerboard tests with the anomaly size of 1.5 km and the interval between anomalies of 0.5 km (contour lines depict the shape of synthetic anomalies). In both cases, the anomaly amplitude is ±9%, but in the first model, the dVp and dVs have opposite signs (a and b), whereas in the second model, the anomalies are equal (c and d). In the models, the anomalies remain unchanged with depth. Note that the size of anomalies in this test is considerably smaller than the structures derived from the inversion of experimental data. The image has been produced using the Surfer Golden Software 13 (https://www.goldensoftware.com/products/surfer).

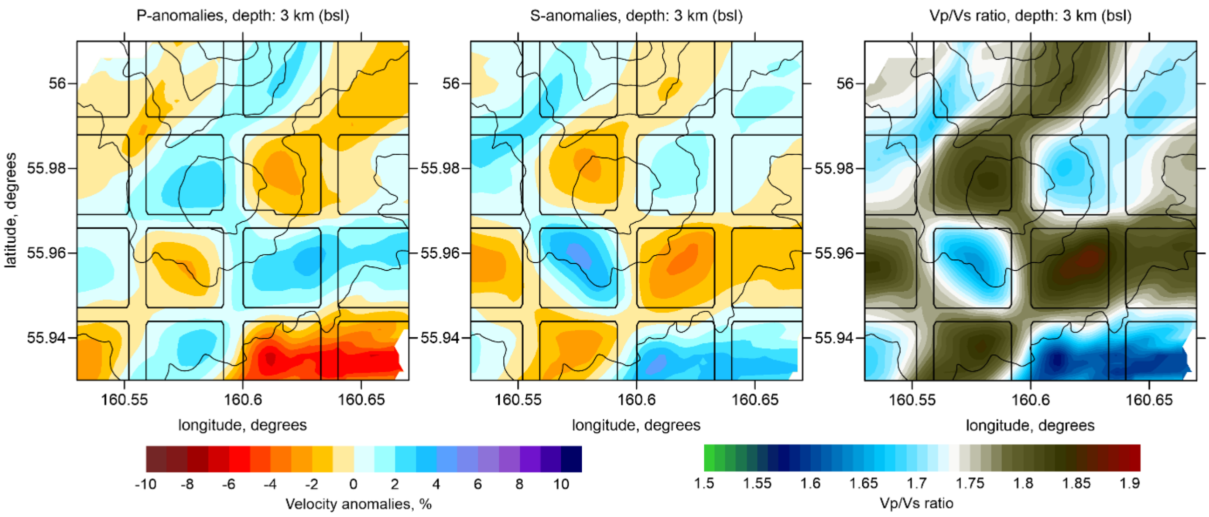

Figure S6. Similar synthetic test as in Figure S5, but in this case, the synthetic anomalies of ±9% are merely defined below the depth of 0.5 km b.s.l. The lateral size of anomalies is 2 km and the interval is 0.5 km. The reconstructed model shows that the existing ray coverage is sufficient to resolve the deep anomalies. The image has been produced using the Surfer Golden Software 13 (https://www.goldensoftware.com/products/surfer).

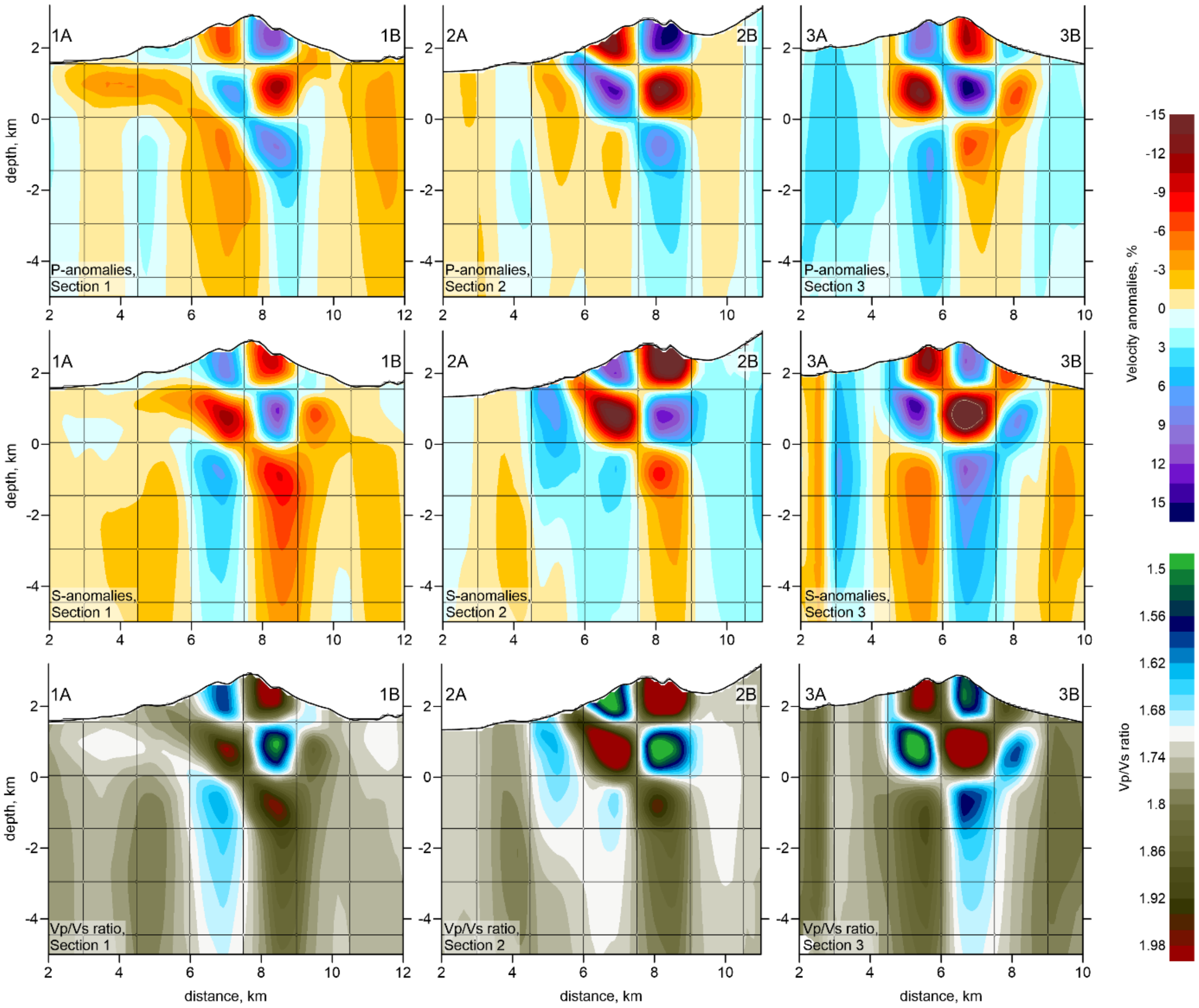

Figure S7. Results of three synthetic tests in which the checkerboard anomalies are defined along each of the three sections used for presenting the main results. The size of anomalies is 1.5 km. Note that the tomography recovers two levels of the sign change at -1.5 km and 0. The deeper anomalies appear to be smeared. The image has been produced using the Surfer Golden Software 13 (https://www.goldensoftware.com/products/surfer).

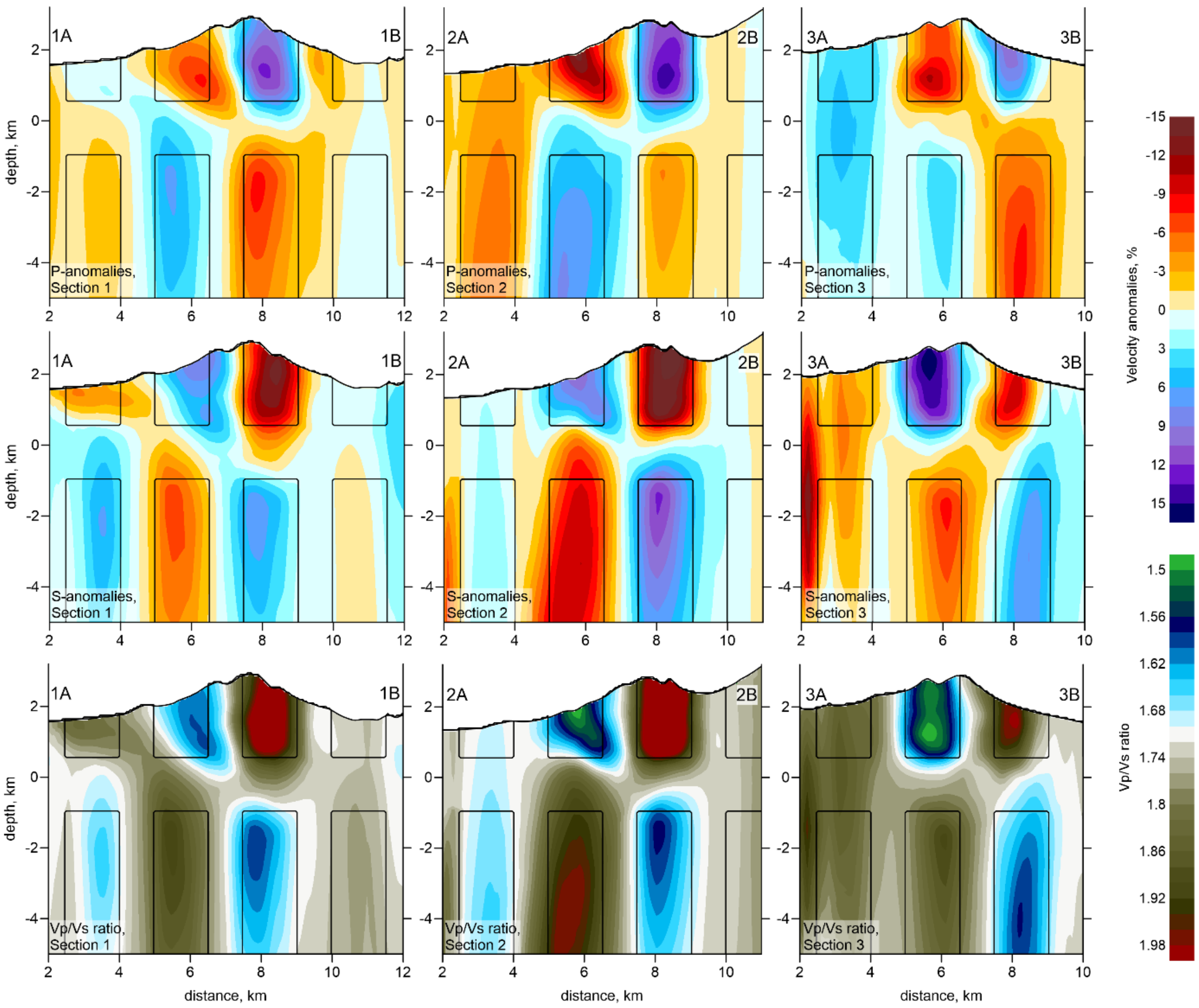

Figure S8. Similar synthetic tests as in Figure S6, but in this case, the transition between shallow and deep synthetic anomalies occurs in the depth interval from -0.5 km to 0.5 km. We can see that both rows of anomalies are recovered beneath the central part of the area. The image has been produced using the Surfer Golden Software 13 (https://www.goldensoftware.com/products/surfer).

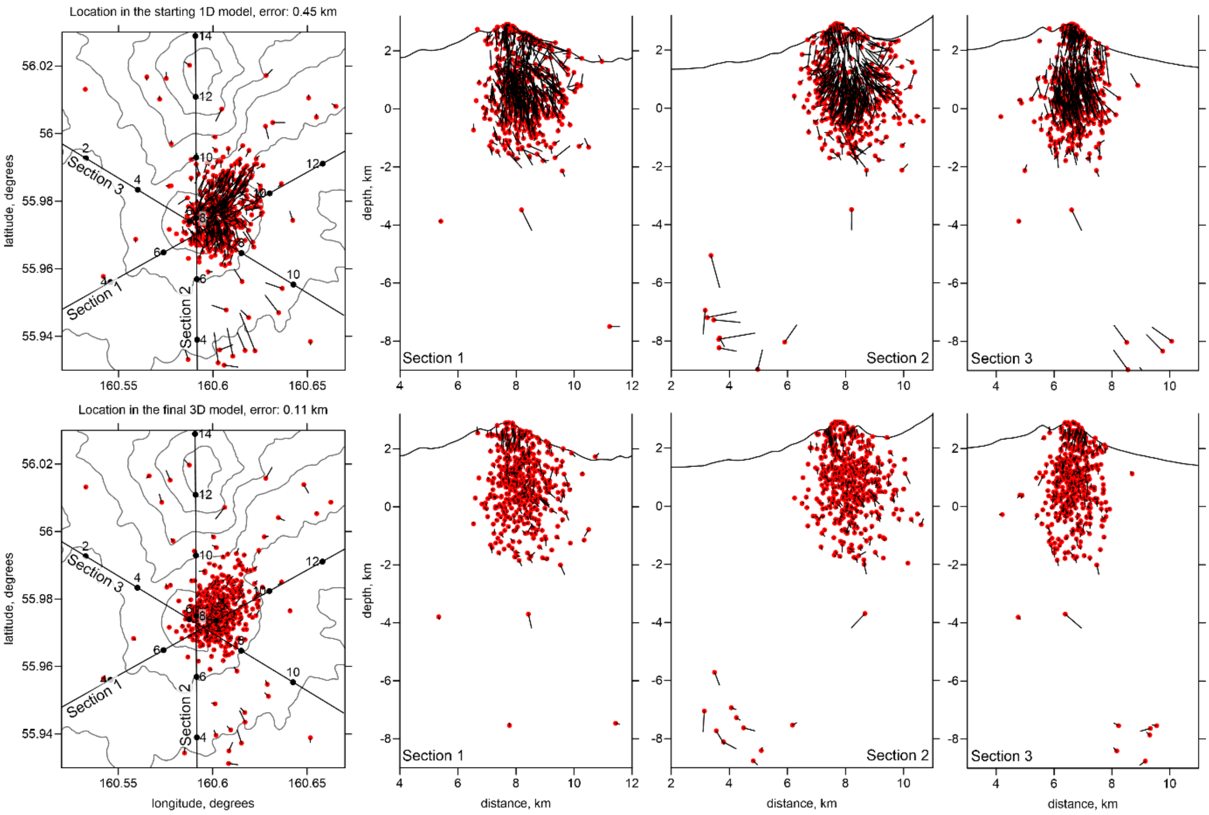

Figure S9. Results of source locations in the case of the synthetic model in Figures S7 in map view and in three vertical sections. Red dots are the resulting source locations; lines indicate to the true locations. Upper row shows the location result in the starting 1D model (error 0.45 km), and the lower row is the location result in the final 3D model (error 0.11 km). The image has been produced using the Surfer Golden Software 13 (https://www.goldensoftware.com/products/surfer).

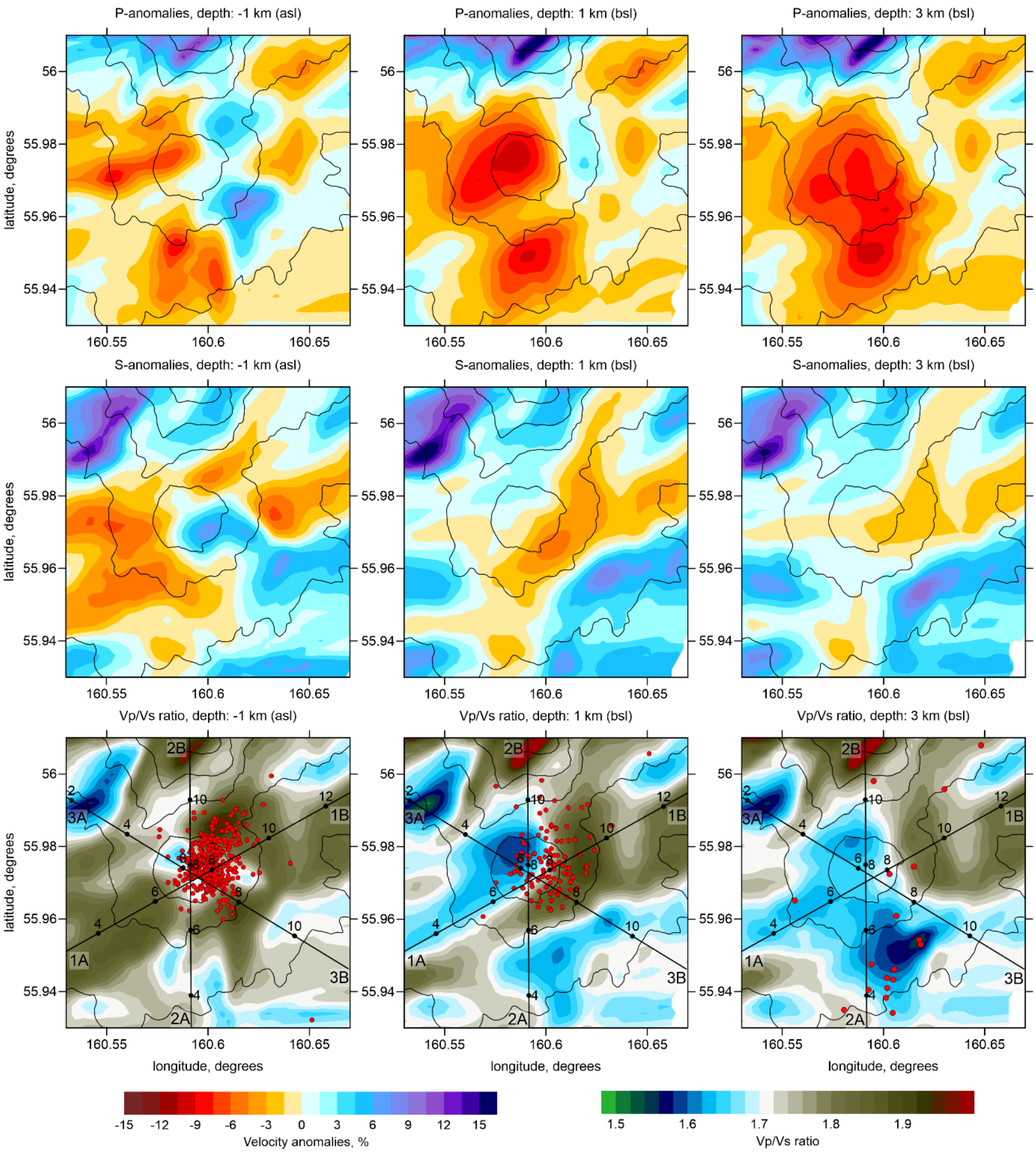

Figure S10. The distributions of *dVp*, *dVs* and *Vp/Vs* ratio in three horizontal sections. The locations of seismic events at the vicinity of the sections are shown with red dots in plots with Vp/Vs ratio. Note that at the shallow depth, beneath the volcano, the high Vp, low Vs and very high Vp/Vs ratio may indicate the presence rigid igneous rocks highly contaminated with liquids (fluids and/or melts). At 3 km depth, we observe very low Vp (“sponge” saturated with gas), normal Vs and low Vp/Vs that may be a signature of gas contaminated rocks. The image has been produced using the Surfer Golden Software 13 (https://www.goldensoftware.com/products/surfer).

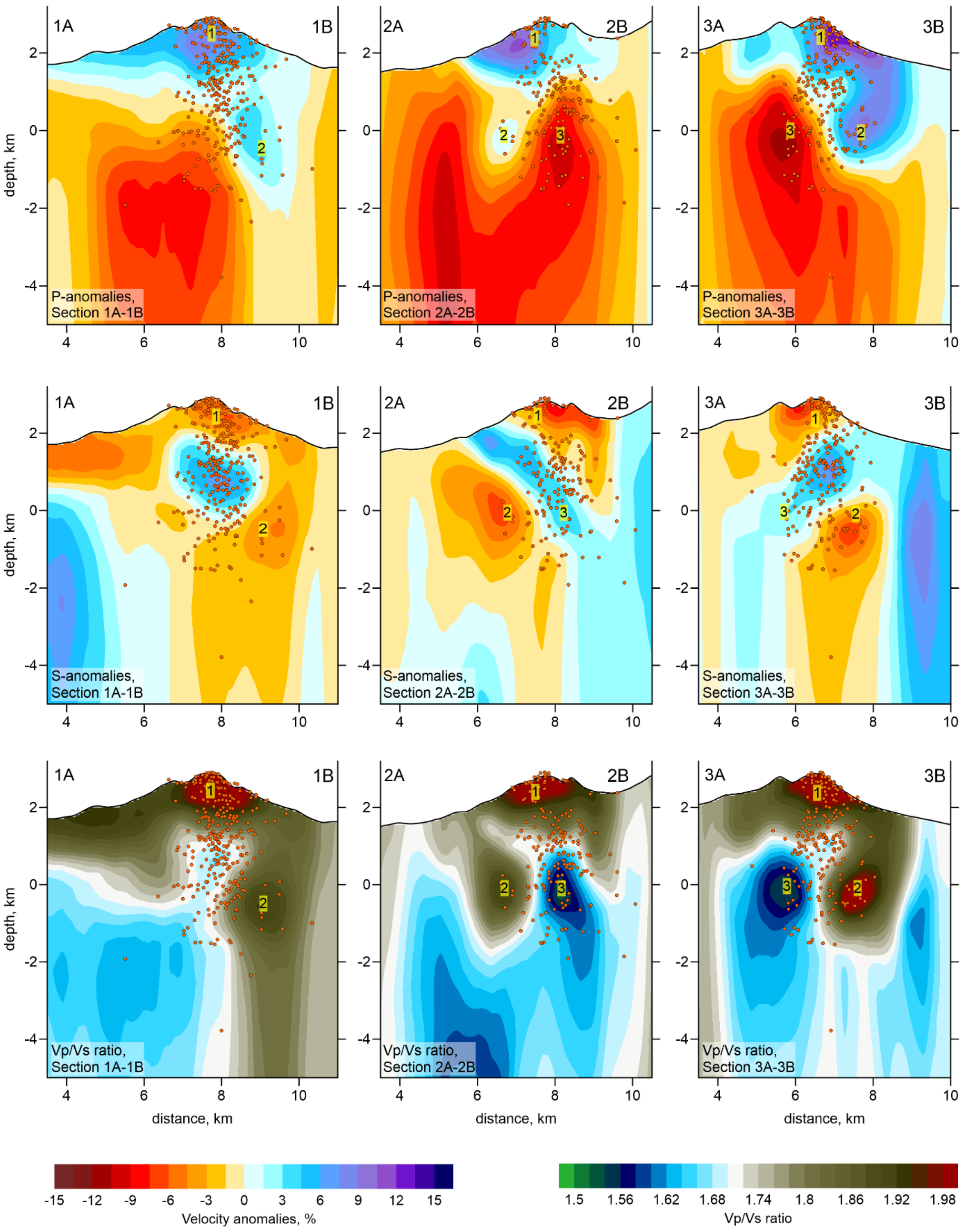

Figure S11. Results of tomographic inversions for the *Vp* and *Vs* anomalies and the *Vp/Vs* ratio in three vertical sections. The locations of the profiles are shown in Figure S9 in the map with the *Vp/Vs* distribution. Red dots indicate the locations of events at the distance of less than 0.5 km from the sections. Numbers indicate the structures discussed in the main paper: 1 – shallow anomaly representing volcano edifice saturated with meteoric waters; 2 – shallow magma reservoir; 3 – gas storage. The image has been produced using the Surfer Golden Software 13 (https://www.goldensoftware.com/products/surfer).

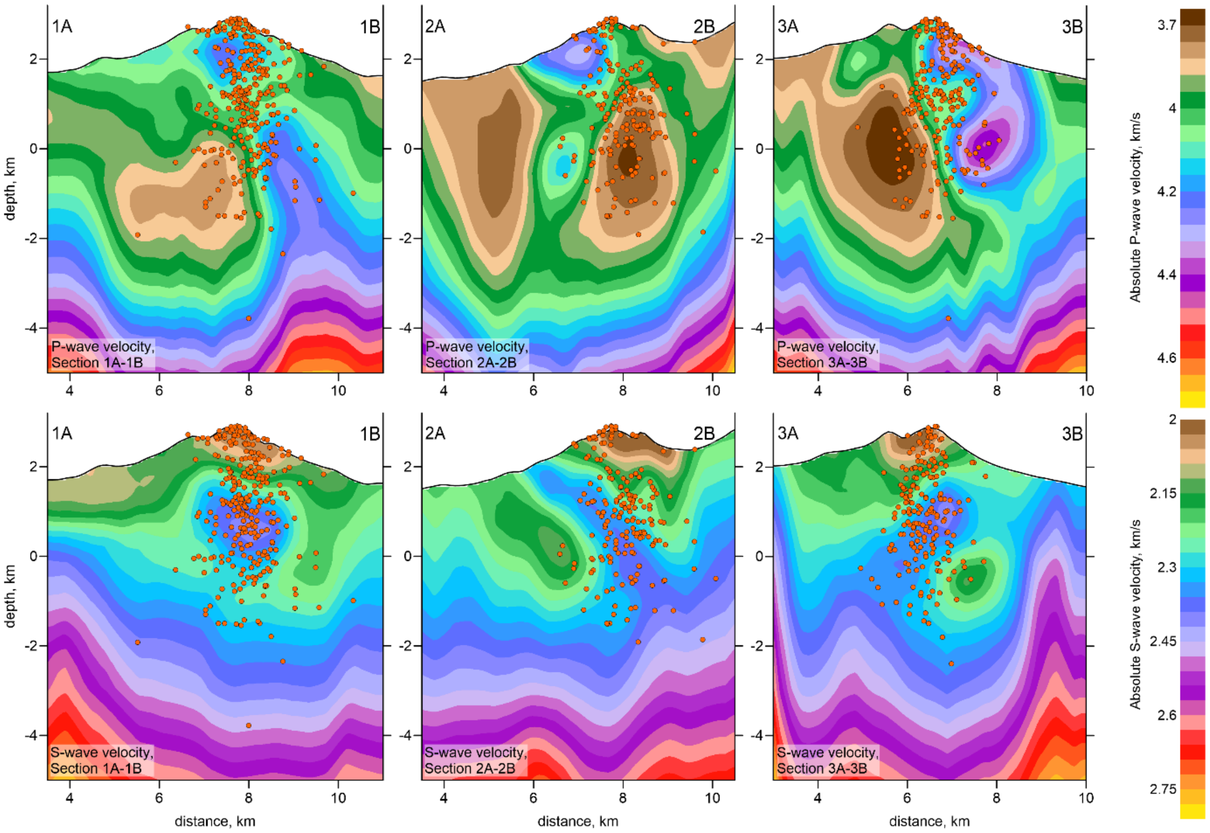

Figure S12. The distributions of absolute values of *Vp*, *Vs* and *Vp/Vs* ratio in three vertical sections. Red dots depict seismic events at a distance of 2 km from the profile. The locations of the profiles are indicated in Figure S9. The image has been produced using the Surfer Golden Software 13 (https://www.goldensoftware.com/products/surfer).

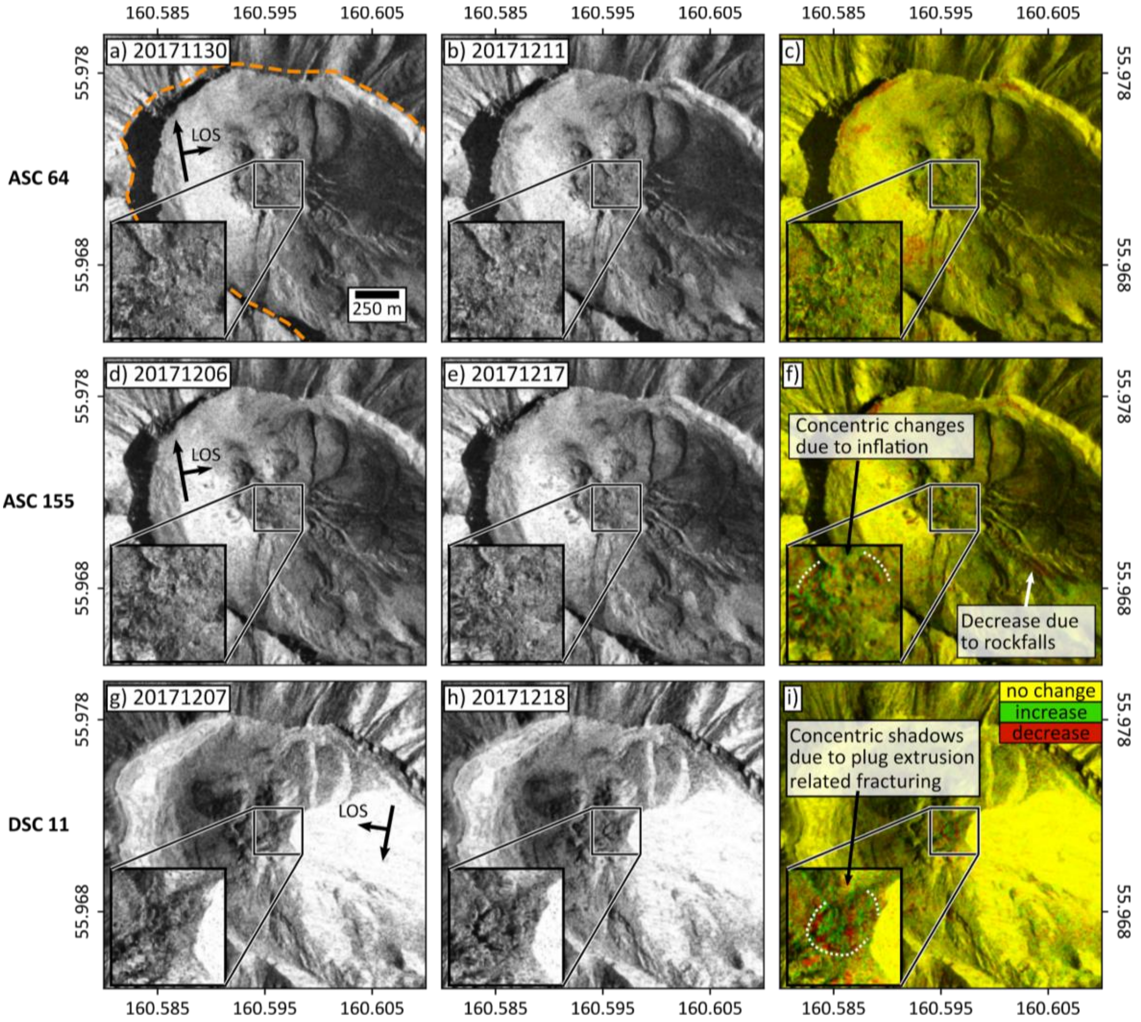

Figure S13. Geocoded TSX amplitude imagery of Bezymianny from the two ascending (ASC) tracks 64 and 155 as well as from the descending (DSC) track 11 (**a, b, d, e, g, h**). Each satellite flight direction and line-of-sight (LOS) is shown in the left panel (blue arrows). Orange dashed line in a) depicts the 1956 collapse scar. The right panel (**c, f, i**) contains the difference image between the images in the same row. Inset maps enlarge the summit area to indicate changes prior to the 20 December 2017 eruption. Please note that until 20171211 (**b**) no obvious changes occurred at the summit, whereas the middle and bottom rows reveal changes likely related to inflation (**f**) and the formation of significant radar shadows caused by either new summit fractures or the extrusion of a new plug. Images are generated using GAMMA Remote Sensing software as well as Matplotlib, OpenCV libraries in Python. TerraSAR-X (TSX) spotlight-mode satellite images available from German Aerospace Center (DLR).
